# Bacterial RecD2 is a processive single-stranded DNA translocase with strand-switching capacity at DNA forks

**DOI:** 10.1093/nar/gkaf459

**Published:** 2025-06-06

**Authors:** Silvia Hormeño, Cristina Ramos, Javier Mendia-Garcia, Clara Aicart-Ramos, Silvia Ayora, Fernando Moreno-Herrero

**Affiliations:** Department of Macromolecular Structures, Centro Nacional de Biotecnología, Consejo Superior de Investigaciones Científicas, 28049, Madrid, Spain; Department of Microbial Biotechnology, Centro Nacional de Biotecnología, Consejo Superior de Investigaciones Científicas, 28049, Madrid, Spain; Department of Macromolecular Structures, Centro Nacional de Biotecnología, Consejo Superior de Investigaciones Científicas, 28049, Madrid, Spain; Department of Macromolecular Structures, Centro Nacional de Biotecnología, Consejo Superior de Investigaciones Científicas, 28049, Madrid, Spain; Department of Microbial Biotechnology, Centro Nacional de Biotecnología, Consejo Superior de Investigaciones Científicas, 28049, Madrid, Spain; Department of Macromolecular Structures, Centro Nacional de Biotecnología, Consejo Superior de Investigaciones Científicas, 28049, Madrid, Spain

## Abstract

RecD2 is a superfamily 1B helicase involved in DNA replication and repair, modulating replication restart, fork progression, and RecA recombinase activity. In this work, we have characterized the functions of *Bacillus subtilis* RecD2 using biochemical and single-molecule approaches. ATPγS binding and low MgCl_2_ concentrations enhance DNA association, with a preference for forked structures and unstructured DNA longer than 30 nucleotides. RecD2 binds to end-less single-stranded DNA stretched at 8–20 pN and translocates through ATP hydrolysis over long distances (>20 kb) with 5′–3′ polarity at high rates. RecD2 shows limited unwinding activity on fork structures, strongly dependent on protein concentration and duplex length, reflecting low processivity. However, processivity improves significantly when force is applied to the translocating strand or unwound DNA ends, enabling the unwinding of thousands of base pairs at rates up to 160 bp/s. Single-molecule assays reveal frequent strand switching on fork substrates, resulting in a non-productive cycle of unwinding and rewinding, likely mediated by the N-terminal domain. This behavior explains the low helicase activity observed in bulk assays. We propose that regulation of strand-switching activity may be relevant for RecD2’s *in vivo* function.

## Graphical Abstract

**Graphical Abstract:**
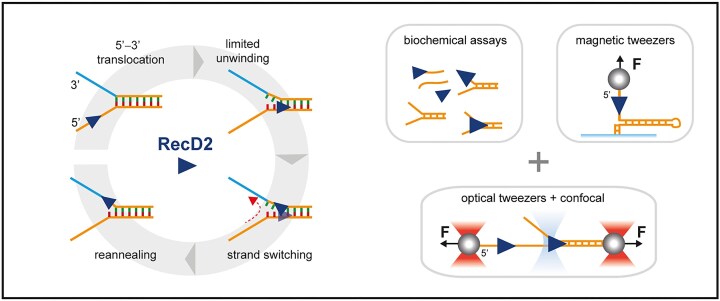

## Introduction

Bacterial RecD enzymes are a group of DNA helicases involved in genome maintenance. They belong to the superfamily 1B (SF1B) of DNA helicases, as they translocate with 5′–3′ polarity [1–4]. RecD enzymes are further divided into two subgroups: RecD1 (known as RecD) and RecD2 [5, 6] (Supplementary Fig. S1A–D). RecD helicases form part of the well-studied RecBCD complex [7], crucial for double-strand break repair, whereas RecD2 helicases are found in bacteria that lack the RecBCD proteins [5, 6, 8, 9]. In *Bacillus subtilis*, RecD2 deletion has been shown to sensitize cells to agents that hinder advance of the replication fork [3]. Moreover, RecD2 is linked to active replication forks through its association with the single-stranded DNA (ssDNA) binding protein SsbA [10] and modulates replication restart as well as homologous recombination by interacting with the RecA recombinase [11]. RecD2 helicases have an extended N-terminal region (NTD) of around 280 amino acids (aa), which remains uncharacterized, although it has been proposed to be involved in DNA binding [9]. Moreover, mutations in conserved motifs located in the NTD of *Bacillus anthracis* RecD2 affect its DNA repair function *in vivo* [9]. The NTD of RecD helicases (110 aa) is shorter than that of RecD2 and is responsible for the interaction with the RecC subunit of the RecBCD complex [12]. The best-studied member of the RecD2 group is *Deinococcus radiodurans* RecD2 (RecD2*_Dra_*), but much of the biochemical data were obtained with a truncated version of RecD2*_Dra_* lacking the first 150 aa (referred to as Δ150-RecD2*_Dra_*) (Supplementary Fig. S1A). Δ150-RecD2*_Dra_* is a monomer in the X-ray structure and translocates following an “inchworm” mechanism with one base movement per hydrolyzed ATP [13, 14]. Despite this structural knowledge, the mechanisms underlying its activity in genome stability remain poorly understood.

Interestingly, RecD2 shares homology with human DNA helicase B (HELB) [15], which is also involved in DNA replication and genome maintenance [16–18]. HELB also has an extended NTD region of around 450 aa, which is not conserved in RecD2 proteins. The function of this domain of HELB remains unclear, but it has been demonstrated to play a significant role in the *in vitro* interaction with CDC45, a component of the CMG replicative helicase [16]. Importantly, a secondary ssDNA binding site has been proposed to be located in this NTD of HELB [19]. Interestingly, AlphaFold 3 modeling of full length RecD2*_Dra_* and RecD2 predicts the presence of an Oligonucleotide/Oligosaccharide-Binding (OB) fold-like motif in the NTD [20]. The OB-fold is a structural motif generally consisting of a five-stranded closed beta-barrel, commonly found in proteins that bind ssDNA across all domains of life [21]. AlphaFold 3 predicts the interaction of this NTD of RecD2 proteins with dT_8_, an 8-mer oligonucleotide of poly(dT) ssDNA (Supplementary Fig. S1E and F).

In this study, we have characterized the activity of full-length RecD2 helicase from *B. subtilis* using bulk and single-molecule assays. Our bulk assays show that the ATPase activity is maximally stimulated as the length of ssDNA with no secondary structures is increased, and single-molecule assays show that RecD2 can translocate at high speeds of 570 nt/s on stretched ssDNA substrates with a high processivity. DNA binding is stimulated by ATPγS and low MgCl_2_ concentrations, with a preference for non-replicated forked structures and unstructured DNA longer than 30 nt. Helicase activity was detected in bulk assays in double-stranded DNA (dsDNA) substrates containing a 5′-ssDNA tail, but this activity was dependent on both the length of the duplex DNA to be unwound and the protein concentration. Notably, the unwinding activity of RecD2 strongly increased when assisted by force in magnetic and optical tweezers experiments, reaching processivities of thousands of base pairs. Interestingly, we found that RecD2 is prone to switch between strands at fork interfaces explaining the limited processivity of duplex unwinding reported in bulk. Our findings shed light on the activity of RecD2 helicases and their potential role in DNA replication and recombination. The implications of these findings are discussed.

## Materials and methods

### Buffers

Buffer A consists of 50 mM Tris (pH 7.5), 40 mM NaCl, 2 mM MgCl_2_, 2 mM Dithiothreitol (DTT), and 0.05 mg/ml Bovine Serum Albumin (BSA). Buffer B contains 50 mM Tris (pH 7.5), 50 mM NaCl, 2 mM DTT, and 0.05 mg/ml BSA. When used, reactions additionally contained the indicated concentration of MgCl_2_ and ATP or ATPγS. To produce single-stranded tethers or ss–dsDNA hybrids, TE buffer (10 mM Tris, pH 8, 1 mM Ethylenediaminetetraacetic acid (EDTA)) is used, as a low-salt environment facilitates the melting of the double helix.

### RecD2 purification and labeling

RecD2 was purified using the protocol described in [11]. The His-tag does not affect protein activity *in vivo* [11], so it was not removed and the purified protein is referred to as RecD2.

The RecD2 protein was labeled with quantum dots (QD) for C-Trap experiments (see below) using a 6x-His Tag monoclonal antibody. The conjugation was carried out following the protocol and reagents provided in the SiteClick™ Qdot 525 antibody labeling kit (Invitrogen). The RecD2 protein was incubated with Qdots-anti-His at a ratio of 1:5 on ice for 30 min in 6 μl. The mixture was then diluted to 200 μl in buffer A and added to the flow cell along with ATP.

### DNA substrates for biochemical assays

Oligonucleotides used in DNA substrate preparation are listed in Supplementary Table S1, with a scheme of substrate composition shown in Supplementary Table S2. As indicated in the figure with an asterisk, one oligonucleotide was 5′-end labeled with [γ-^32^P]ATP and T4 polynucleotide kinase. In strand-switching assays, Cy5-labeled oligonucleotides were used. Annealing reactions, containing 100 mM Na-phosphate buffer (pH 7.5), the fluorescent or radiolabeled oligonucleotide, and the necessary unlabeled oligonucleotides (1*:2 ratio), were heated at 95°C for 5 min and then slowly cooled to 25°C over 3 h. Substrates were purified by electrophoresis and elution from the gel using TE buffer, incubated overnight at 4°C, ethanol-precipitated, and resuspended in TE buffer. DNA concentrations in binding and helicase assays are expressed in moles of molecules, while in ATPase assays they are expressed in moles of nucleotides.

### Electrophoretic mobility shift DNA binding assays

Standard binding reactions had 0.25 nM DNA radiolabeled DNA, buffer B, 1 mM ATPγS, and variable concentrations of MgCl_2_ and RecD2. Reactions were incubated 15 min at 37°C. Glutaraldehyde (0.05%, v/v) was added to stabilize protein–DNA complexes and samples were incubated another 15 min at 37°C. Protein–DNA complexes were separated by 8% polyacrylamide gel electrophoresis (PAGE) in TBE 0.5× and visualized by autoradiography. Complexes were quantified using the Image Lab software (Bio-Rad).

The apparent binding constant (*K*_app_) of the RecD2–DNA complexes—defined as the protein concentration at which 50% of the DNA is bound—was determined by plotting the data using Excel.

### ATPase assays

The ssDNA-dependent ATPase activity of RecD2 was analyzed using a spectrophotometric assay that coupled ATP and Nicotinamide Adenine Dinucleotide (NADH). The effector DNA (3 μM in nucleotides) was pre-incubated for 5 min at 37°C with 5 nM RecD2 in buffer B, which contained 0.5 mM phosphoenolpyruvate, 0.3 mM NADH, 10 U/ml pyruvate kinase, 10 U/ml lactate dehydrogenase, and varying concentrations of MgCl_2_. After incubation, 0.5 mM ATP was added and reactions were further incubated for 250 s at 37°C. The decrease in NADH absorbance at 340 nm was monitored to determine the rate of ATP hydrolysis. The rate was calculated by measuring the slope of the linear portion of each absorbance scan and is expressed as [ATP in μM]/[RecD2 in μM]/s. *V*_max_ and *K*_m_ data values were obtained from fitting data to the Michaelis–Menten equation in Prism 10 program.

### Helicase assays

The helicase activity of RecD2 was analyzed using radiolabeled or Cy5-labeled DNA. The reaction mixture consisted of 0.25 nM radiolabeled DNA or 3 nM fluorescent DNA, buffer B, 1 mM ATP, 2 mM MgCl_2_, and varying concentrations of RecD2. The reactions were incubated for 15 min at 37°C and then stopped by adding a stop buffer (0.8 mg/ml Proteinase K, 20 mM EDTA, 0.5% sodium dodecyl sulfate, 10% glycerol, 0.1% bromophenol blue, and 400 nM of unlabeled oligo, added to trap the displaced strand and prevent reannealing of the labeled oligos). The products were separated by 15% PAGE and visualized using the PMI Imager and Chemidoc Touch Imaging systems (Bio-Rad). The image was then quantified using Image Lab software (Bio-Rad).

### DNA substrates for single-molecule experiments

#### ssDNA substrate for C-Trap experiments

The design of an ssDNA construct of 22 261 nt employed to study the translocation activity of RecD2 was fabricated as described in [22].

#### Hybrid ssDNA–dsDNA for C-Trap experiments

The design of a hybrid ssDNA–dsDNA construct of 17 303 bp was fabricated as detailed in [22]. In this particular case, the central part of the DNA construct was obtained by digesting the described large homemade plasmid that contained a unique BbvCI restriction site with XhoI and NotI (New England Biolabs). Without further purification, the fragment was ligated with T4 DNA ligase (New England Biolabs) to two highly biotinylated handles of ∼1 kb ending in PspOMI or XhoI, being the XhoI handle dephosphorylated by rSAP (New England Biolabs). This created a nick in the lower strand. After the ligation step, the dsDNA molecules were digested with the nicking enzyme Nt.BbvCI (New England Biolabs) for 2 h at 37°C followed by 20 min at 80°C for heat inactivation. This led to a final DNA construct with two nicks in the same strand, to facilitate the process of removal of the lower strand by pulling in the optical tweezers setup, to create a gap of 2319 nt. The sample was ready for use without further purification. DNAs were never exposed to intercalating dyes or UV radiation during their production and were stored at 4°C.

#### DNA hairpin for magnetic tweezers experiments

The design of a DNA hairpin construct consisting of a 1238-bp hairpin with a 4-nt loop (4 dTs), a 31 bp 5′ and 3′-biotinylated labeled dsDNA tail followed by a 45-nt ssDNA segment, and a 146 bp 3′-digoxigenin labeled dsDNA tail was based on a previously published construct with slight modifications [23]. The 45-nt ssDNA segment between the 31-bp biotinylated labeled dsDNA tail and the dsDNA central part of the hairpin is long enough for RecD2 protein to bind and has the proper orientation (5′–3′) for the protein to translocate toward the dsDNA central part of the hairpin. Detailed process of fabrication is described in Supplementary data, including oligonucleotides used in the DNA construct preparation (Supplementary Table S3) as well as the sequence of the DNA hairpin (Supplementary Table S4).

### Optical tweezers and confocal microscopy assay

We employed a dual-trap optical tweezers combined with confocal fluorescence microscopy (C-Trap) from Lumicks, B.V. A multi-channel fluidics cell was used for the experiments, which is depicted in Supplementary Fig. S2A. The experiment began by trapping two streptavidin-coated polystyrene beads (4.38 μm, Spherotech) in two 1064 nm laser traps in channel 1. The trapped beads were transferred to channel 2, where a biotinylated DNA tether was attached between the two beads. To confirm that only a single tether with the expected length was successfully trapped, or to produce the final functional DNA construct, the DNA-tethered beads were subsequently moved to channel 3. To investigate RecD2 activity with the C-Trap, we have employed two distinct DNA samples: a dsDNA construct of 22.3 knt that can be melted *in situ* to generate a full-length ssDNA molecule, and a hybrid single-stranded (2.3 knt) and double-stranded (15 kb) DNA construct [22]. The fully or partially ssDNA tethers were created by stretching the initial construct beyond (Supplementary Fig. S2B) or to the onset (Supplementary Fig. S2C) of the overstretching transition, respectively. This force-induced melting procedure was carried out in channel 3 containing TE buffer. Finally, the functional DNA constructs were moved to channel 4, previously passivated with 0.1% (w/v) BSA, where the proteins were loaded and imaged. To visualize the activity of RecD2 labeled with QDs, we performed constant-force experiments illuminating with a 488 nm excitation laser (blue). Kymographs were generated by means of confocal line scans between the center of the two beads (50 nm pixel size and ∼50 ms per line scan).

Force, extension, and fluorescence data were analyzed using custom-written Python scripts and LabVIEW programs. We used Origin 2021 for statistical analysis and distribution fittings. RecD2 translocation velocity was directly obtained from the slope of the linear trajectories using FIJI [24]. To calculate the instantaneous unwinding velocity of RecD2, we analyzed unwinding traces using linear regression of the extension along segments located between pauses. This approach enabled us to determine the maximum rate of unwinding of RecD2 at a given force.

To accurately determine the relationship between the measured distance and the number of translocated nucleotides or unwound base pairs, we conducted experiments characterizing the mechanical properties of a dsDNA and an ssDNA molecule under the same conditions in which RecD2 activity was measured (Supplementary Fig. S3A).

### Magnetic tweezers setup

The magnetic tweezers setup employed in this study was based on designs previously described in the literature [25, 26]. It features a pair of vertically aligned NdFeB permanent magnets (Supermagnete), separated by a 0.3 mm gap, with a flow chamber placed above an inverted microscope. The flow chamber was created by sandwiching a top coverslip, containing two small holes for inlet and outlet, with a double-sided adhesive (Adhesive Research, 92712) that forms a 5 mm wide channel, and a lower coverslip coated with polystyrene creating a channel with ≈10 μl volume. DNA hairpins linked to superparamagnetic beads were introduced into the chamber and immobilized on the bottom surface. The permanent magnets positioned over the flow chamber enabled manipulation of the tethered DNA molecules by attracting the beads. The beads’ movement was tracked at 120 Hz using an inverted microscope equipped with a high-magnification oil-immersion objective and a CCD camera. The force acting on the DNA molecules was determined based on the distance between the magnets and the chamber, calculated from the molecules’ Brownian motion [26]. The extension of the DNA molecules was measured by comparing images at varying focal planes. This setup allowed the simultaneous monitoring of up to 25 molecules and the application of forces up to 20 pN on 1 μm beads.

The DNA hairpins were anchored to the surface and the superparamagnetic beads using the following procedure: the flow chamber was incubated overnight at 4°C with 100 ng/μl of Digoxigenin Antibody (Bio-Rad) to allow passive adsorption to the polystyrene-coated lower surface. Prior to the experiment, the chamber was passivated with PBS–BSA (1 mg/ml BSA in Phosphate-Buffered Saline (PBS), New England Biolabs) for 45 min. For the DNA-bead preparation, 0.3–0.5 ng of DNA in TE buffer was combined with 5 μl of 1 μm magnetic beads (Dynabeads MyOne Streptavidin C1, Invitrogen) at stock concentration, previously cleaned in PBS–BSA. The mixture was incubated on a rotator for 10 min at room temperature, after which 25 μl of PBS–BSA was added and incubated for another 20 min. During this period, the biotinylated ends of the DNA attached to the streptavidin-coated magnetic beads. Then, 5–10 μl of this mixture was added to 20 μl of PBS–BSA and introduced into the passivated flow cell, where the digoxigenin-labeled DNA end bound to the antibodies on the lower coverslip. After 8 min, excess beads were removed by flushing with RecD2 reaction buffer A.

### Magnetic tweezers hairpin assay

Magnetic tweezers were used to pull on a 1238-bp hairpin (Supplementary Tables S3 and S4) in an unzipping configuration. One end of the DNA molecule was tethered to the lower glass surface and the other one to a superparamagnetic bead. The hairpin was kept at forces at which it remained closed (8–14 pN), and a 50 pM concentration of RecD2 in buffer A supplemented with 1 mM ATP was flown into the chamber. The substrate has a 45-nt ssDNA region where the protein can be loaded and start unwinding. The advance of the protein in the 5′–3′ direction results in the unwinding of the dsDNA hairpin region and an increase of extension. Unwinding rates were calculated as linear regressions of this part of the time course. Once the protein unwinds the whole dsDNA region and reaches the hairpin loop, it can continue its way by translocating in the ssDNA it has in front. Given that in the absence of protein at the applied forces, the hairpin is closed, RecD2 translocation can be followed by looking at the decrease of extension caused by hairpin closure behind the protein. Translocation rates were calculated as linear regressions of this part of the time course.

ssDNA force extension curves were obtained to get the nt/nm equivalence in the RecD2 reaction buffer. For this, we used a blocking oligonucleotide strategy as described in [27]. Briefly, the hairpin is mechanically opened by applying a force above 16 pN, and then a 400 nM solution of a 28 nt-long oligonucleotide (5'-GGCATCTGACCCGAGCACTACTGGCTGG) is flowed into the cell. This oligonucleotide is complementary to a sequence of the hairpin located close to its loop. The oligonucleotide acts as kinetic barrier, blocking hairpin rezipping above 1–2 pN forces. This permitted the acquisition of ssDNA force–extension curves in buffer A.

Unwinding and translocation traces resulting from protein activity were filtered by applying a nonlinear filter [28] with parameters *K* = 40, *M* = 5, and *P*= 1 two times. Then, linear regressions were conducted for each unwinding/translocation event to obtain the unwinding and translocation velocities. The unwinding and translocation rates were calculated for forces between 8 and 14 pN.

### AlphaFold 3 models

The full protein structures of RecD2 proteins from *B. subtilis* and *D. radiodurans* were obtained from the AlphaFold Protein Structure Database. To generate DNA-bound models, the AlphaFold server was employed. The structures of the NTDs of *B. subtilis* (residues 1–325) and *D. radiodurans* (residues 1–329) in complex with an 8-nt polythymidine oligonucleotide sequence (dT_8_) were modeled. Model 0 was selected from the proposed structures for NTDs.

For the structure of *B. subtilis* RecD2 bound to a forked DNA substrate, the sequences of two oligonucleotides (5′-TTTTTTTTTTTTTTTCCCCCCCCCCCCCCC-3′ and 5′-GGGGGGGGGGGGGGGTTTTTTTTTTTTTTT-3′) were considered alongside the sequence of *B. subtilis* RecD2. Among the proposed models, the one closest to that of DNA-bound *D. radiodurans* RecD2 described in [13] was selected.

### Statistical methods

The biochemical data were analyzed using Excel and Prism 10 (GraphPad) software. The results were represented as mean ± SD (standard deviation). Statistical differences between samples were assessed using the non-parametric Mann–Whitney test. Single-molecule data were analyzed using OriginPro software.

## Results

### Full-length BsRecD2 is an ssDNA-dependent ATPase

The ATPase activity of the RecD2*_Dra_*, the RecD2 protein from *D. radiodurans*,has been previously characterized [13, 29], with most studies focusing on the N-terminal truncated version of the protein (Δ150-RecD2*_Dra_*). This truncated protein exhibited minimal variation in ATPase rates with poly(dT) substrates of different lengths (20–60 nt), maintaining an average rate of ∼100 s^−1^ [13]. The ATP hydrolysis activity of the full-length RecD2*_Dra_* was only evaluated using a 17-mer oligonucleotide in the presence of 10 mM MgCl_2_, yielding a rate of 9.0 ± 0.3 s^−1^ [4]. No further studies have analyzed the ATPase activity of other RecD2 helicases. To expand our understanding of RecD2 helicase function, we sought to investigate the impact of both the nature and length of ssDNA effectors on the ATPase activity of full-length RecD2 from *B. subtilis* (hereafter, just RecD2) and to determine the optimal magnesium concentration for these reactions in bulk assays.

**Figure 1.**
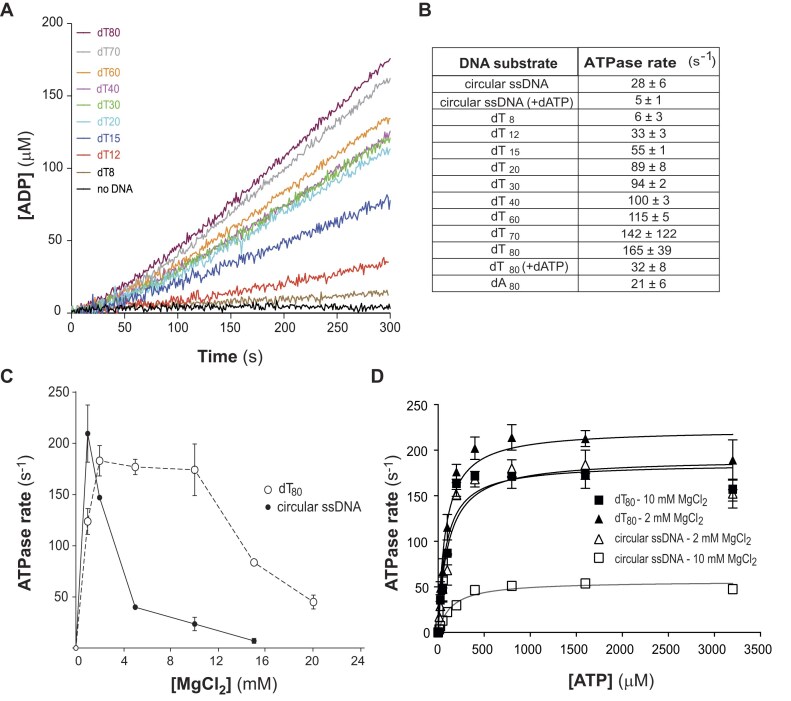
ssDNA-dependent ATPase activity of RecD2. (**A**) ATPase activity in the presence of different ssDNAs. Reactions containing 5 nM RecD2 and the indicated ssDNA cofactor (3 μM in nucleotides) were incubated at 37°C in the presence of 10 mM MgCl_2_. The level of ATP hydrolysis was measured by the spectrophotometric coupled ATP/NADH assay. Plots are the mean of three independent experiments. (**B**) Average ATPase rate determined from the linear slope of the curves with different ssDNA as effectors. (**C**) Effect of MgCl_2_ concentrations on the ATPase activity of RecD2 in the presence of dT_80_ or circular ssDNA [sspGEM3Zf (+)]. (**D**) Michaelis–Menten plot of ATP hydrolysis for RecD2 in the presence of circular ssDNA or dT_80_ as cofactors and 2 or 10 mM MgCl_2_. The kinetic parameters obtained are listed in the text. The data shown are the averages of three assays, and the errors represent the SD.

As previously reported for RecD2*_Dra_* [4], the ATPase activity of RecD2 was stimulated by the presence of ssDNA in our experiments (Fig. 1A), but the activity was almost undetectable with a dT_8_ ssDNA and was low with dT_12_ or an ssDNA containing secondary structures [circular ssDNA, sspGEM-3Zf (+)] (Fig. 1A and B). RecD2 activity was further assessed using poly(dT)s of varying lengths (15–80 nt) without secondary structures. The results showed a low rate of 55 ± 1 s^−1^ with dT_15_ and a moderate increase in ATPase activity with poly(dT)s ranging from 20 to 60 nt.

Further increases in ATPase activity were observed with dT_70_ (142 ± 12 s^−1^) and dT_80_ (165 ± 39 s^−1^), suggesting that ATPase activity increases with the length of unstructured ssDNA. The activity with circular ssDNA and dT_80_ was also tested using dATP, resulting in significantly lower dATPase rate of 32 ± 8 s^−1^ for dT_80_ and 5 ± 1 s^−1^ for circular ssDNA. Interestingly, ATP hydrolysis was poorly stimulated by dA_80_ (Fig. 1B). A possible explanation for this may be the particular elasticity of poly(dA) structure, which has been reported to be strongly influenced by base-stacking interactions [30].

The impact of varying Mg^2+^ concentrations was also assessed. The ATPase rate showed minimal variation when different MgCl_2_ concentrations (between 2 and 10 mM) were applied to unstructured ssDNA (dT_80_). However, the results were significantly different when using circular ssDNA, likely because the Mg^2+^ favors the stabilization of secondary structures in circular ssDNA (Fig. 1C). To further explore the effect of Mg^2+^ concentration, Michaelis–Menten constants for ssDNA substrates with and without secondary structures were calculated (Fig. 1D). The kinetic data for dT_80_ showed similar *K*_m_ and *V*_max_values at both Mg^2+^ concentrations:*K*_m_= 82 μM ATP at 2 mM MgCl_2_ and 85 μM ATP at 10 mM MgCl_2_ with *V*_max_ of ∼223 and 185 s^−1^, respectively. The activity of RecD2 with circular ssDNA at 2 mM MgCl_2_ was comparable to that with dT_80_, with a *K*_m_ of 107 μM ATP and a *V*_max_ of *∼*190 s^−1^. However, at 10 mM MgCl_2_, a slight decrease in affinity was observed for circular ssDNA, with a *K*_m_ of 145 μM ATP and a significantly reduced *V*_max_ of 56 s^−1^ (Fig. 1D).

Together, we found that the ATPase activity of RecD2 was stimulated by ssDNA and increased with longer poly(dT) substrates. The presence of secondary structures or higher Mg^2+^ concentrations reduced RecD2 activity, especially with structured ssDNA [sspGEM-3Zf (+)]. Kinetic analysis showed similar *K*_m_ values for dT_80_ between 2 and 10 mM MgCl_2_, while circular ssDNA exhibited decreased affinity and a significantly reduced *V*_max_ at higher Mg^2+^ concentrations. These results indicate that RecD2 prefers longer, unstructured ssDNA and is sensitive to both DNA structure and magnesium concentration.

### RecD2 binds to unstructured ssDNA regions larger than 20 nucleotides

The interaction of RecD2 proteins with various DNA substrates has been poorly explored. Hence, we investigated DNA binding using electrophoretic mobility shift assays (EMSAs). First, to determine the optimal conditions for the stability of protein–DNA complexes, we used an 80-nt ssDNA with a random sequence. Experiments were performed both in the presence and in the absence of 1 mM ATPγS, a non-hydrolyzable ATP analogue, as well as in the presence or absence of the crosslinker glutaraldehyde. The results showed that binding affinity was not affected in the absence of glutaraldehyde, but the protein–DNA complexes did not form well-defined bands (Supplementary Fig. S4A and B). Notably, ATPγS increased binding by ∼4–8-fold (Supplementary Fig. S4C). Consequently, all subsequent EMSA experiments were conducted using 1 mM ATPγS and 0.05% (v/v) glutaraldehyde added after a 15-min incubation period prior to electrophoresis.

**Figure 2.**
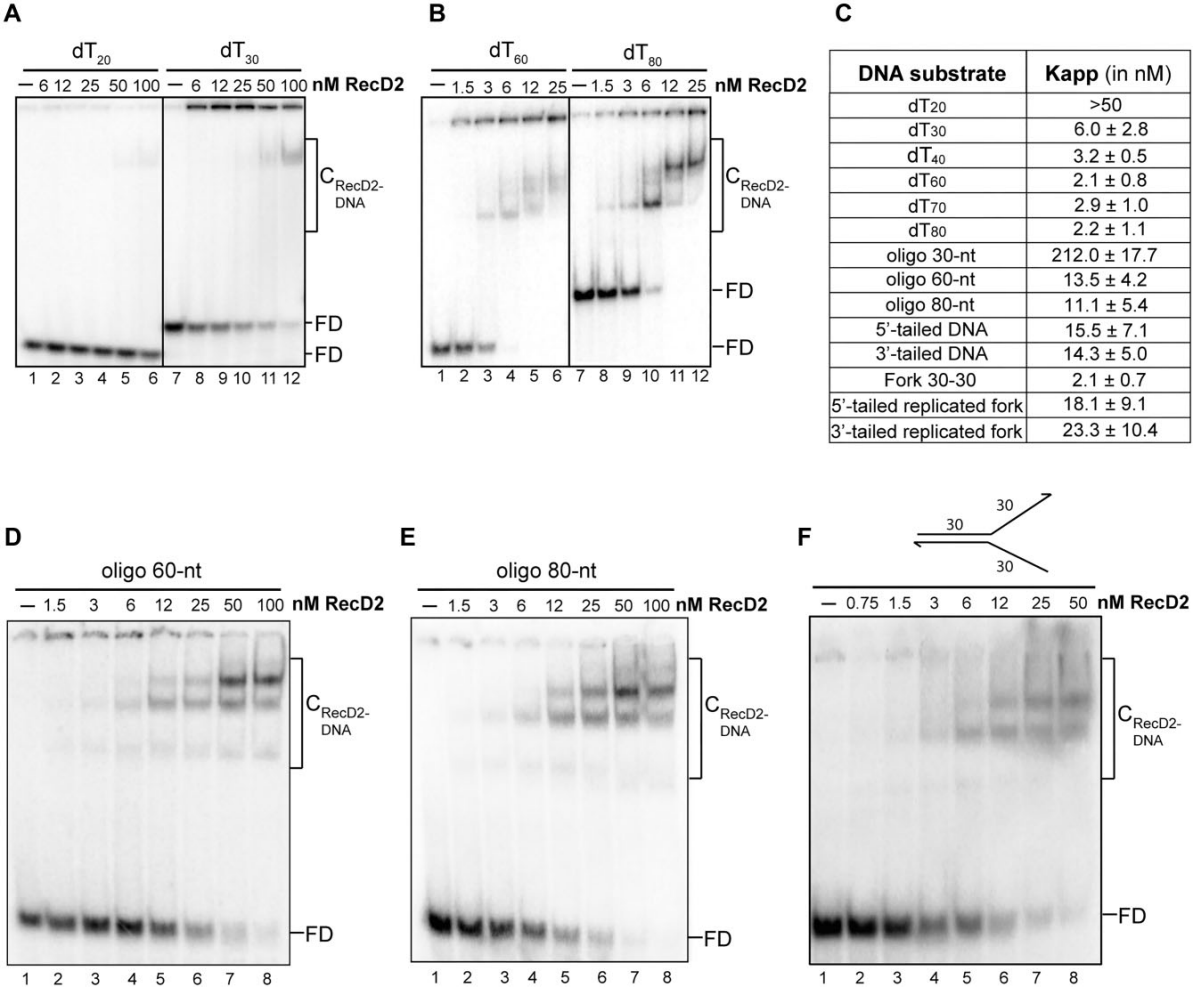
DNA binding of RecD2. Increasing amounts of RecD2 were incubated for 15 min at 37°C with radiolabeled ssDNA (0.25 nM in molecules) in a binding buffer containing 2 mM MgCl_2_ and 1 mM ATPγS. Then, glutaraldehyde (0.05% v/v) was added, samples were incubated for another 15 min, and then separated on 8% PAGE run in 1× TBE. DNA substrates: (**A**) dT_20_ (lanes 1–6) and dT_30_ (lanes 7–12); (**B**) dT_60_ (lanes 1–6) and dT_80_ (lanes 7–12). (**C**) Summary table with apparent binding constants *K*_app_ (nM), which represent the concentration of RecD2 that binds 50% of DNA, shown as mean ± SD for at least three independent experiments for all substrates assayed (data from this figure and Supplementary Figs S5 and S6): (**D**) 60-nt ssDNA, (**E**) 80-nt ssDNA, and (**F**) fork 30–30. FD: free DNA; C: protein–DNA complexes.

Next, we examined how substrate length and the ability to form secondary structures influence RecD2 binding. First, we investigated the effect of DNA length on RecD2 binding using unstructured ssDNA. RecD2 binding affinity was low for dT_20_ (*K*_app_ > 50 nM) but increased significantly with dT_30_, with a *K*_app_ value of around 6 nM (Fig. 2A and C). Affinity continued to increase as the length of the poly(dT) increased, with *K*_app_ values of ∼3 ± 1 nM for poly(dT) lengths ranging from 40 to 80 nt (Fig. 2B and Supplementary Fig. S5A and B). Consistent with our previous findings, RecD2 exhibited low binding affinity to a 30-nt ssDNA with a random sequence, with a *K*_app_ value >200 nM (Fig. 2C and Supplementary Fig. S5C). However, the affinity improved markedly when the length of the structured ssDNA increased to 60 nt (*K*_app_ = 13.5 ± 4.2 nM) (Fig. 2D) and 80 nt (*K*_app_ = 11.1 ± 5.4 nM) (Fig. 2E). Additionally, in line with prior ATPase assays, RecD2’s binding affinity to structured substrates was lower at 10 mM MgCl_2_ compared to 2 mM MgCl_2_ (Supplementary Table S5). Interestingly, at a length of 60 nt, three distinct RecD2–ssDNA complexes were observed in the gels for both structured and unstructured DNAs (Fig. 2B and D), indicating the binding of multiple helicases to a single ssDNA molecule. These results suggest a binding size around 20 nt and provide evidence for cooperative binding of multiple RecD2 molecules to the DNA, because lower mobility complexes appeared before all the ssDNA substrate was shifted. Additionally, these assays demonstrated that RecD2 preferentially binds to unstructured ssDNAs.

RecD2 has been proposed to associate with replication forks [10]. To investigate this, we evaluated RecD2’s affinity for fork-like and tailed substrates *in vitro* using EMSA assays. RecD2 exhibited similar affinities for both 5′- and 3′-tailed structures of 30 nt, with *K*_app_ values of 16 ± 7 and 14 ± 5 nM, respectively (Fig. 2C and Supplementary Fig. S6), indicating no specific preference for a 5′ ssDNA tail and that the presence of an ss–ds junction enhances the binding, since the affinity to these substrates was 14 times better than to the 30-nt oligonucleotide (*K*_app_ ∼15 nM versus 212 nM). Furthermore, RecD2 displayed a very high binding affinity for an unreplicated fork structure with 30-nt tails (fork 30–30), with a *K*_app_ value of 2 ± 1 nM, suggesting a strong preference for this replication intermediate (Fig. 2C and F). As to ssDNA, the binding to the fork was stabilized by glutaraldehyde (Supplementary Fig. S7). These results showed that the presence of an additional ssDNA arm in the proximity enhances the binding, perhaps because the helicase contacts the two arms of the fork. The 5′-arm is bound through the region identified in the RecD2–ssDNA crystal structure [13], and the 3′-arm of the fork through an additional binding site, perhaps in the NTD domain (see later). To analyze this further, we compared the binding affinity to the replication fork with the affinity to partially replicated forks that do not have two ssDNA arms but only one. The affinity to these structures decreased and was similar to the affinity for tailed substrates, confirming that RecD2 contacts also the other ssDNA arm of the fork (Fig. 2C and Supplementary Fig. S6). Together these results suggest that the preferred substrate for the RecD2 helicase will be a region of ssDNA with no secondary structure and longer than 30–40 nt or a non-replicated fork.

### RecD2 is a 5′–3′ helicase with unwinding activity on 16-nt tailed fork structures

**Figure 3.**
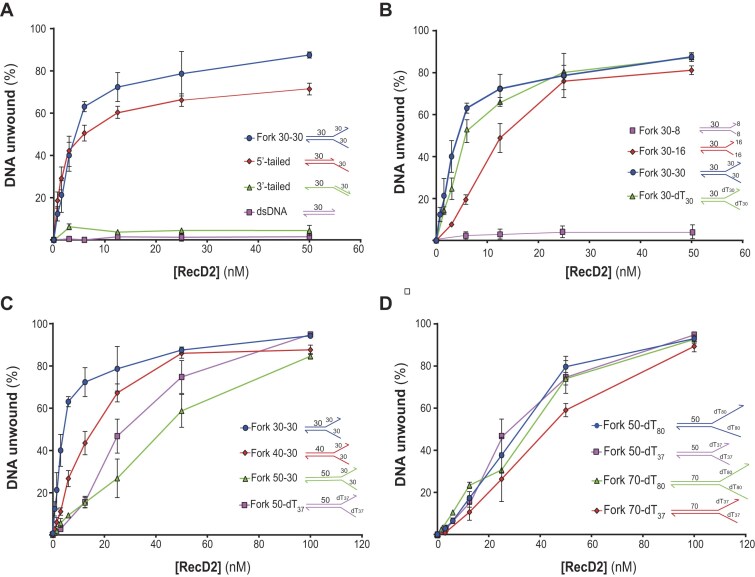
Analysis of RecD2 DNA unwinding activity from bulk assays. In all assays, 0.25 nM DNA molecules of radiolabeled structures were incubated with increasing concentrations of RecD2 (0.75–100 nM) for 10 min at 37°C. Then, reactions were stopped and separated on 10% PAGE in 0.5× TBE buffer. The percentage of DNA unwound (%) was quantified from the gels by the ImageLab software. (**A**) RecD2 unwinds 5′-tailed and forked DNA. The substrates used are fork 30–30 (blue), 5′-tailed (red), 3′-tailed (green), and dsDNA (pink). (**B**) The effect of the tail length and structure in the unwinding of forked structures. The substrates used are fork 30–8 (pink), fork 30–16 (red), fork 30–30 (blue), and fork 30-dT_30_ (green). (**C**) Unwinding activity of RecD2 with longer dsDNA regions. Helicase assays were performed with the radiolabeled fork structures: 30–30 (blue), 40–30 (red), 50–30 (green), and 50-dT_37_ (pink). (**D**) Long tails do not further increase the unwinding of forked structures. Substrates used: 50-dT_80_ (blue), 50-dT_37_ (pink), 70-dT_80_ (green), and 70-dT_37_ (red). The graphs represent the mean ± SD of at least three independent experiments for each substrate, and representative gels are shown in Supplementary Fig. S8.

We evaluated RecD2’s DNA unwinding ability using bulk assays, monitoring the displacement of a ^32^P-labeled oligonucleotide using PAGE. Our results confirmed that RecD2 is a 5′–3′ helicase, requiring a DNA duplex with a 5′-single-stranded tail for activity, as no unwinding was observed for a 30-bp duplex with blunt ends or a 3′-ssDNA tail (Fig. 3A and Supplementary Fig. S8). Consistent with previous findings on RecD2*_Dra_*, RecD2 unwound 5′-tailed and a fork 30–30 structure with similar efficiency (Fig. 3A and Supplementary Fig. S8) [4].

To further analyze the effect of the ssDNA tail length and composition, we tested four different ^32^P-labeled forked substrates with identical 30-bp duplex regions but varying the tail length with random sequence (fork 30–8, fork 30–16, and fork 30–30) or with poly(dT) (fork 30-dT_30_) (Fig. 3B and Supplementary Fig. S8). Consistent with the low ATPase activity found with dT_8_, no helicase activity was detected with this substrate. The fork 30–16 substrate was less efficiently unwound, especially at low protein concentrations, consistent with RecD2’s lower affinity for shorter oligonucleotides. However, there was no significant difference in helicase activity between fork 30–30 and fork 30-dT_30_.

To investigate RecD2’s processivity, we tested forked substrates with 30, 40, and 50 bp duplexes and 30 nt 5′ and 3′ tails. At 25 nM RecD2, the fork 30–30 and fork 40–30 substrates were effectively unwound, while only 27% of fork 50–30 was unwound (Fig. 3C). Substituting the 30-nt random tails with 37-nt poly(dT) tails (fork 50-dT_37_), unwinding activity was only slightly increased, with 50% unwound at the same protein concentration.

Finally, we examined the impact of longer poly(dT) tails by extending them to 80 nt, given RecD2’s higher ATPase activity with dT_80_ compared to dT_30_ (Fig. 1B). We constructed forks 50-dT_80_ and 70-dT_80_ and compared their unwinding activity with forks 50-dT_37_ and 70-dT_37_, respectively. However, extending the tail length to 80 nt did not significantly enhance the unwinding activity (Fig. 3D).

Together, these experiments show that RecD2 is a 5′–3′ helicase, capable of unwinding forked structures if the 5′ ssDNA tails are longer than 16 nt, and showing little activity on duplexes with 8-nt tails, blunt ends, or 3′ tails. However, the unwinding efficiency was affected by the length of the duplex DNA. These results support previous studies that suggested that RecD2 proteins have low processivity during unwinding [4, 31].

### RecD2 translocates with high processivity on unstructured ssDNA

**Figure 4.**
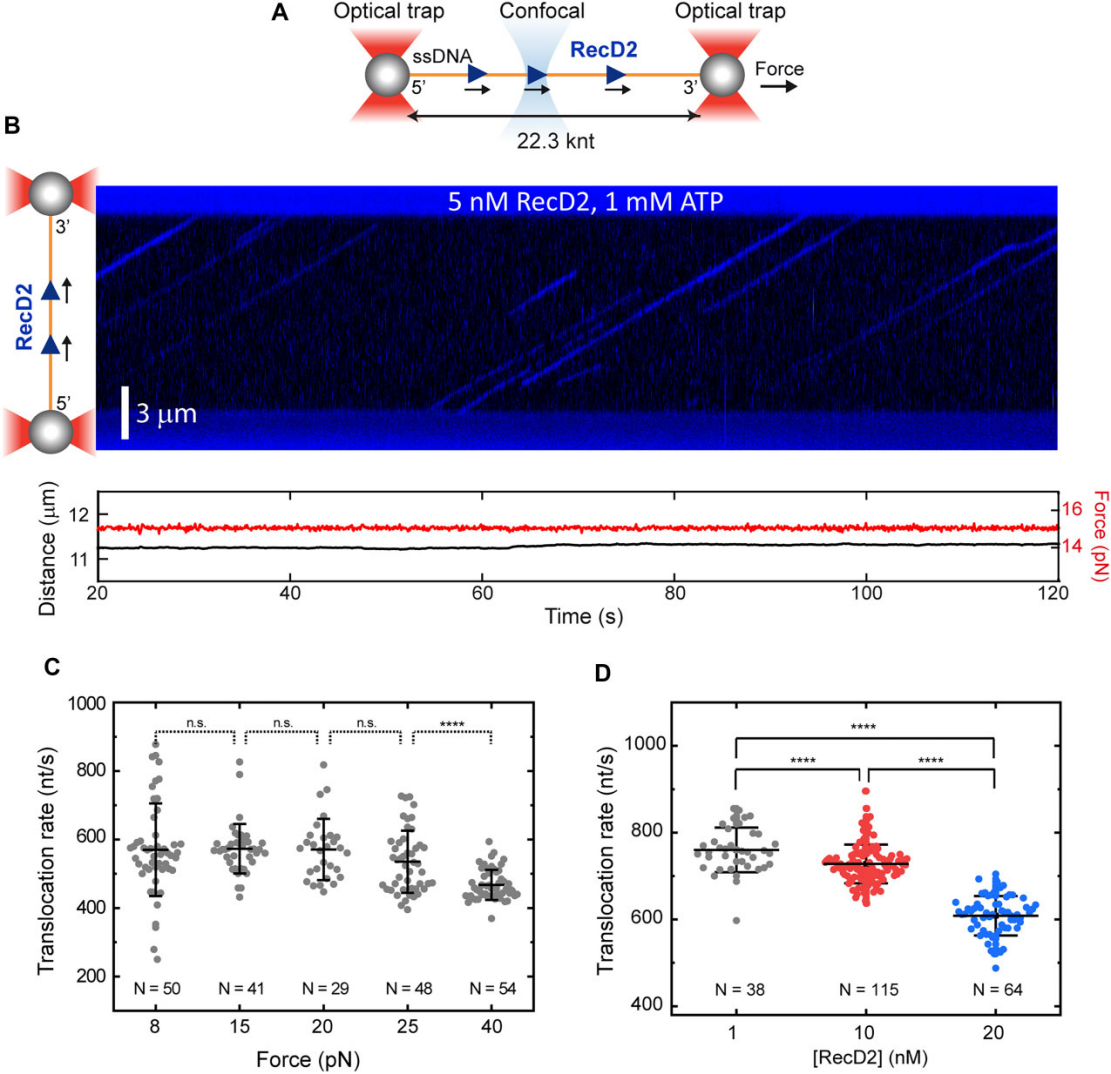
RecD2 is a fast ssDNA translocase with high processivity. (**A**) Illustration of the experimental setup to measure the translocation activity of RecD2 in the C-Trap. Individual 22.3-knt ssDNA tethers were attached between two streptavidin-coated beads trapped by two optical traps. A confocal laser scanned the DNA tethers to detect RecD2 conjugated to one quantum dot. (**B**) Representative kymograph of RecD2 movement (blue) in the presence of 1 mM ATP under 15 pN of tension. No changes in DNA extension are detected (lower panel). (**C**) Interval scatter plots of RecD2 translocation rates on ssDNA as a function of force in the presence of 1 mM ATP. The central bar represents the mean of the data, and the error bars represent the SD. The number of individual trajectories measured at each force is indicated below the data. Statistical analysis revealed no statistical significance of the mean rates at forces ranging from 8 to 25 pN (*P* >0.05). (**D**) Interval scatter plots for RecD2 at different protein concentrations in the presence of 1 mM ATP. The tension on the DNA was kept constant at 15 pN. The central bar represents the mean of the data, and the error bars represent the SD.

To gain deeper insight into RecD2 translocation abilities, we monitored the movement of individual RecD2 proteins along ssDNA using optical tweezers combined with confocal scanning microscopy (C-Trap, Lumicks) [32–35] (Fig. 4A). In these experiments, we used a 22.3-knt ssDNA tether generated by force-induced melting of a duplex DNA construct (Supplementary Fig. S2A). This construct featured a nick in one end due to ligation to a dephosphorylated handle and three nicks along the same strand, one of them being near the intact handle at the opposite end [22] (Supplementary Fig. S2B). The ssDNA tether was then moved to a channel containing the RecD2 protein in buffer A, supplemented with 1 mM ATP (see the “Materials and methods” section). To track the protein’s position, we labeled His-tagged RecD2 with anti-His-Qdot™ 525 dyes and confirmed that both labeled and unlabeled proteins exhibited similar ATPase activity in bulk assays (Supplementary Fig. S9A). The binding and movement of labeled RecD2 proteins along a ssDNA molecule were then visualized by confocal imaging between the beads at ∼50 ms line^−1^, and the resulting kymographs were analyzed. All experiments were conducted under force-clamp conditions at the indicated force.

In the presence of ATP, RecD2 proteins bound the ssDNA and translocated unidirectionally at a uniform velocity (Fig. 4B). As confirmed later through additional single-molecule assays, the proteins likely moved toward the 3′ end, consistent with previous bulk experiments, which showed no unwinding activity on 3′-tailed substrates (Fig. 3A) and aligned with earlier studies from Δ150-RecD2*_Dra_* [13]. Notably, the unidirectional translocation observed in the optical tweezers experiments was initiated without requiring a free DNA end (Fig. 4B). We recorded the trajectories of over 220 RecD2 molecules under varying forces, from 8 to 40 pN, and applied linear regression to each trajectory to determine the average translocation rate (Fig. 4C). The mean velocity for RecD2 translocation was 572 ± 106 nt/s (*N* = 120), with no significant variation in the populations collected at 8, 15, 20, and 25 pN. However, at 40 pN we observed a decrease in translocation rate, likely due to ssDNA distortion under high force, which could disrupt the coupling between ATP hydrolysis and single-nucleotide translocation. Increasing the protein concentration resulted in slower trajectories overall (Fig. 4D), suggesting that protein crowding negatively impacts translocation and does not support the idea that RecD2 oligomerization enhances its activity. Notably, we did not observe DNA loop formation, as reported for human HELB [19], likely due to structural differences between the proteins, particularly in their NTDs, where a secondary DNA binding site for HELB has been proposed [19]. In the absence of ATP, we did not observe RecD2 binding to ssDNA in our single-molecule assays (Supplementary Fig. S9B). This contrasts with biochemical assays where RecD2 was capable to binding ssDNA, albeit with lower affinity than with ATPγS (Supplementary Fig. S4C) and in the presence of glutaraldehyde. As expected, ATPγS impaired RecD2 movement in single-molecule experiments but allowed the stable binding of the protein to the ssDNA (Supplementary Fig. S9C and D). Our results demonstrate that RecD2 is a highly processive enzyme, capable of translocating over thousands of nucleotides on ssDNA free of secondary structures at rates of hundreds of nucleotides per second.

### RecD2 unwinds duplex DNA when assisted by force

**Figure 5.**
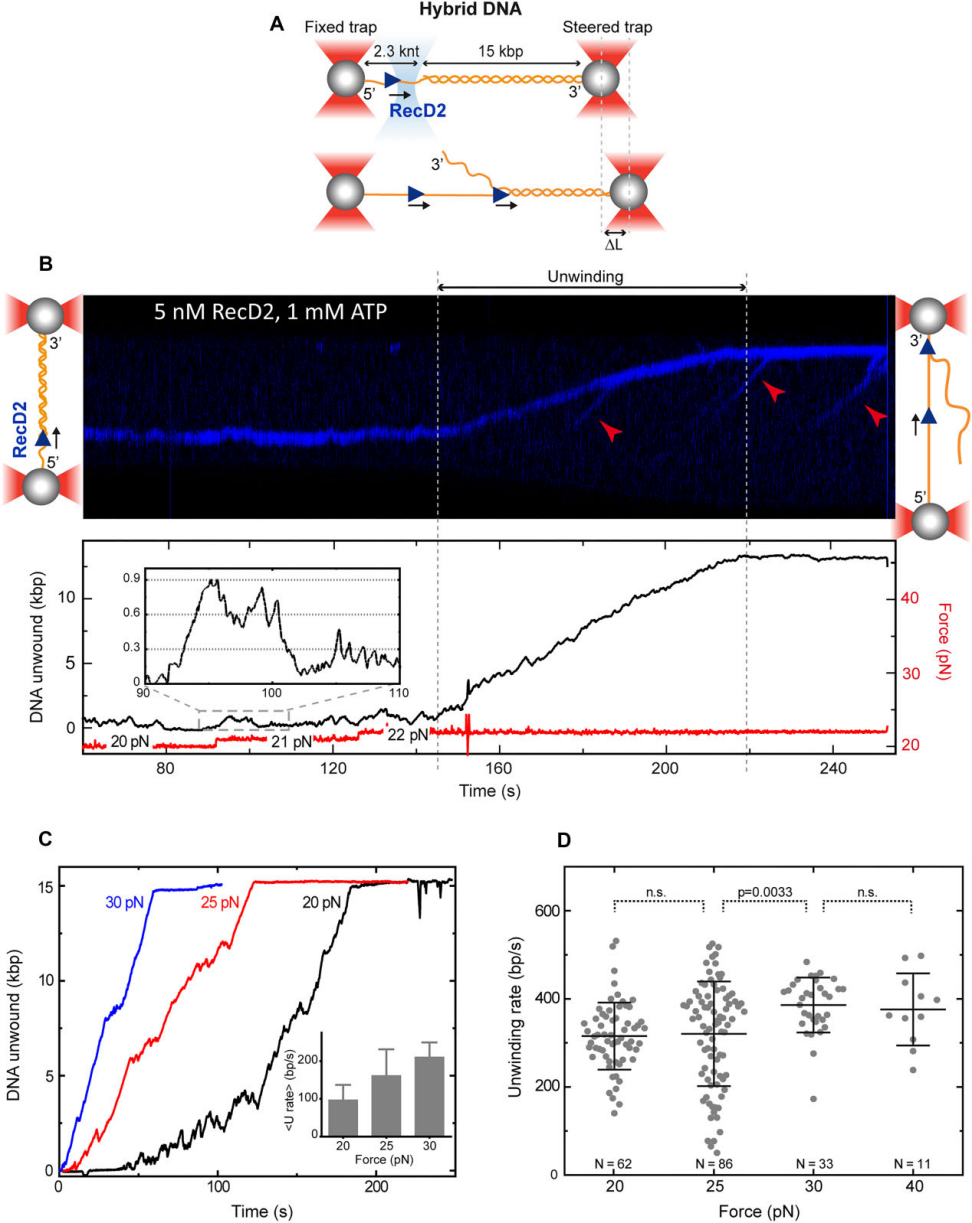
RecD2 unwinds duplex DNA only if assisted by force. (**A**) Illustration of the experimental setup to measure the unwinding activity of RecD2 in the C-Trap. Individual 5′-tailed long DNA tethers (2.3-knt ssDNA and 15-kb dsDNA) were attached between two streptavidin-coated optically trapped beads. A confocal laser scanned the DNA tethers to detect QD-labeled RecD2. (**B**) Representative kymograph of RecD2 activity (blue) in the presence of 1 mM ATP on a hybrid DNA, with force increased stepwise from 20 to 21 and 22 pN. At 22 pN, the unwinding of the double helix by RecD2 is detected as an increment in DNA extension (lower panel). Inset: Extension fluctuations characterized by unwinding and rewinding of short DNA segments. Red arrows indicate translocating RecD2. (**C**) Representative unwinding traces at different forces. The force assists in the unwinding by reducing the number of pauses and increasing the overall unwinding rate (inset, *N* = 17). Note that in all the cases, RecD2 unwinds the complete 15-kb dsDNA fragment of these hybrid substrates. (**D**) RecD2 unwinding rates as a function of force. Each data point corresponds to the slope measured by linear regression of discrete segments along single RecD2 unwinding traces. The central bar represents the mean unwinding rate, and the error bars represent the SD. The total number of data points considered is included in the graph. Statistical analysis revealed no statistical significance of the unwinding rates at 20–25 and 30–40 pN (*P* >0.05).

Previous bulk experiments showed that RecD2 functions as a 5′–3′ helicase, capable of unwinding both 5′-tailed DNA and forked structures, albeit with moderate efficiency (Fig. 3). To investigate RecD2’s unwinding activity at the single-molecule level, we utilized a hybrid ssDNA–dsDNA construct. This construct mimics the ss–dsDNA junction found at replication forks and resembles the 5′-tailed DNA used in the bulk assays, but is significantly longer and lacks a free 5′ end. To produce these substrates, we optically trapped a 17.3-kb dsDNA molecule engineered with two nicks on the same strand and separated by 2.3 kb (Supplementary Fig. S2C) [22]. The hybrid ss–dsDNA tether was created *in situ* by applying a force of ∼50 pN, which was sufficient to partially overstretch the double helix under low-salt buffer conditions, melting the 2.3-knt region between the two nicks. This resulted in a hybrid DNA substrate consisting of a 2.3-knt ssDNA gap and a 15-kb dsDNA region. The tether was then moved to the protein channel, where the activity of labeled RecD2 was monitored (Fig. 5A). We focused on forces above 6 pN, since at these forces, the transition from dsDNA to ssDNA leads to an increase in tether extension (Supplementary Fig. S3A). Therefore, under constant force conditions, RecD2-mediated unwinding should result in the separation of the optical traps.

We observed that RecD2 bound to the ssDNA region and translocated toward the ss–dsDNA junction, confirming its inherent 5′–3′ polarity. However, RecD2 appeared to stall at the ss–dsDNA junction, unable to proceed with unwinding (Fig. 5B). Upon closer inspection of the DNA extension traces, we found that RecD2 was not static but instead engaged in cycles of unwinding and rehybridization of the DNA helix (Fig. 5B, inset, and Supplementary Fig. S10). At 20 pN, RecD2 was able to unwind several hundred base pairs, surpassing the unwinding length observed in bulk experiments. The recovery of the initial DNA extension, indicating helix rehybridization, could result either from the helicase losing its grip on the DNA, leading to rapid rezipping of the helix, or from a strand-switching mechanism. The fact that the rewinding occurred at a rate comparable to that of the unwinding suggests that the protein itself regulated the pace of the rewinding process. RecD2 was able to interact with and translocate along the opposite strand. What causes the helicase to stall during unwinding and to switch strand remains unclear, as the point of return seemed to occur stochastically in our experiments. In the next section, we provide an in-depth analysis of this phenomenon using a magnetic tweezers approach.

The primary determinant influencing RecD2 unwinding activity in these experiments was the tension applied to the DNA substrate. By gradually increasing the force, we found that RecD2’s full unwinding activity was triggered at forces above 22 ± 4 pN (*N* = 15) (Supplementary Fig. S11). Remarkably, once initiated, RecD2 consistently unwound the entire dsDNA segment (∼15 kb). The applied force also affected the unwinding dynamics: higher forces resulted in fewer pauses and fewer backward steps during the unwinding process (Fig. 5C). This suggests that increased force facilitates RecD2 activity, leading to faster overall reaction velocity (Fig. 5C, inset). In order to quantify the RecD2’s helicase rate (i.e. strand unwinding and separation), we calculated the rate of unwinding by fitting the linear sections of the unwinding trajectories between pauses. RecD2’s unwinding rate was 330 ± 100 bp/s at 20–25 pN (*N* = 151) and 400 ± 60 bp/s at 30–40 pN (*N* = 44) (Fig. 5D), which is lower than its translocation rate. As expected, we often observed other labeled RecD2 molecules binding to the ssDNA region behind the leading protein and translocating along the newly created ssDNA (Fig. 5B, arrows). These molecules translocated on ssDNA at similar rates to those reported in the previous section.

### RecD2 is highly prone to strand switching

**Figure 6.**
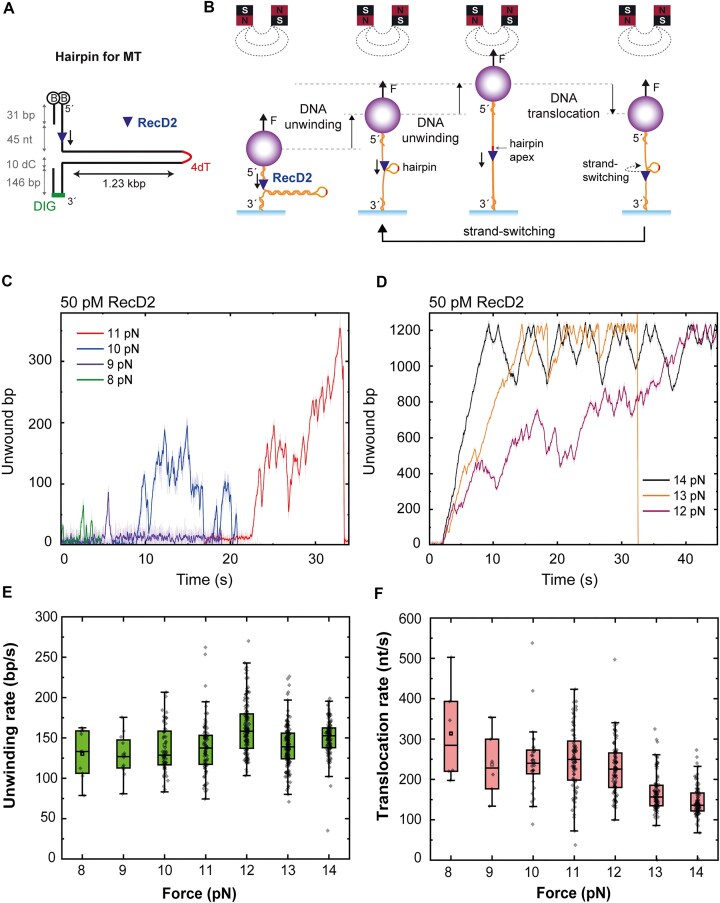
RecD2 exhibits strand switch at ss–dsDNA junctions. (**A**) Schematic representation of the DNA hairpin substrate used in the magnetic tweezers experiment. (**B**) Illustration showing the interpretation of the time courses obtained in MT experiments. RecD2 is loaded in the ssDNA region adjacent to the hairpin and unwinds the dsDNA until it reaches the hairpin loop. Then, it translocates on ssDNA for a short time before switching strands returning to the unwinding mode. (**C**) Representative time courses of the activity of RecD2 in the DNA hairpin at low forces. At forces below 12 pN, RecD2 often fails to fully unwind the hairpin. (**D**) Representative time courses of the activity of RecD2 in the DNA hairpin at higher forces, where RecD2 typically succeeded in fully unwinding the hairpin, but strand-switching events occur. (**E**) Unwinding rate of RecD2 as a function of the applied unzipping force. (**F**) Translocation rate of RecD2 as a function of the applied unzipping force.

To get further insight into the strand-switch phenomenon observed in our previous optical tweezers experiments, we implemented a complementary approach based on magnetic tweezers and a DNA hairpin. In this assay, a DNA hairpin with a duplex length of 1.23 kb was tethered by its ends to a magnetic bead through a 45-nt segment followed by a 31-bp region and to the glass surface through a 10-nt segment followed by a 146-bp region (Fig. 6A). Force–extension curves of the substrate in the absence of the protein showed that the hairpin opened rapidly at 15 pN (Supplementary Fig. S3B). As a consequence, we conducted experiments at constant force below this threshold, so any signal of unwinding was unequivocally due to enzyme activity, detected as an increase of the distance between the bead and the surface (Fig. 6B). Note, however, that once RecD2 reaches the tip of the hairpin, this can re-form immediately behind the motor. Further translocation of the helicase would then cause reannealing of the hairpin in its wake, leading to a reduction of the extension of the tether, as it has been observed for other helicases [36] (Fig. 6B).

In our assays, no activities were detected below 8 pN, likely due to either the protein failing to load onto the hairpin or being unable to unwind it. Between 8 and 10 pN, unwinding events rarely resulted in the complete opening of the hairpin (Fig. 6C). Interestingly, these events typically ended not because the protein detached from the hairpin but because it switched strands. This switch changed protein activity from unwinding to translocation, leading to hairpin closure behind the protein. To obtain further evidence of strand switching by RecD2, we performed biochemical assays. We prepared several Cy5-fluorescent forks with different lengths of the 3′-arm of the fork and keeping constant the 5′-arm. As observed with the radiolabeled substrate, the protein was not able to unwind a 5′-tailed substrate with a tail of 8 nt, nor a fork with 8-nt tails (Fig. 3B and Supplementary Fig. S12A). Increasing the length of the 3′-arm to 16 nt resulted in 40% of the substrate unwound at 400 nM RecD2, and a further increase of the length of the 3′-arm to 30 nt resulted in the highest unwinding activity (Supplementary Fig. S12A). These results suggested that a 3′-arm at an ss–ds junction can assist in the loading of the helicase to the neighboring 5′-arm, facilitating the strand-switching mechanism.

At forces above 10–11 pN, RecD2 could unwind the whole hairpin. However, in this case, once the protein passed the hairpin apex, a brief decrease of extension, corresponding to translocation, was followed by an increase of extension, consistent with hairpin opening (Fig. 6D). This process occurred cyclically, generating a saw-like time–position trace until the protein eventually detached from the hairpin, at which point the extension instantly decreased to the close-hairpin position. We interpreted this behavior as a continuous cycle of unwinding, translocation, and strand switching (Fig. 6B). This type of activity has already been described for other helicases such as RecQ [37] or the SF1B helicase, Pif1 [38]. We detected strand-switching events not only when the helicase had reached the hairpin apex but also at intermediate stages of hairpin opening (Fig. 6C), though these became less frequent as force increased (Fig. 6D). Interestingly, similar experiments performed with a higher RecD2 concentration (20 nM) results in a full opening of the entire hairpin at 8 pN force, which was not possible at pM protein concentrations. This is consistent with the cooperative opening by multiple proteins shown by Ha and Lohman for the UvrD helicase [39] (Supplementary Fig. S12B).

Overall, across the range of forces tested (8–14 pN), the unwinding rate of RecD2 exhibited consistency, with variations within a narrow band between 128 and 162 bp/s (Fig. 6E). These rates were significantly higher than those reported in [31] for RecD2*_Dra_* (69 bp/s) using MT and a similar hairpin assay. Notably, we observed an increase in RecD2 translocation rate, and concomitantly hairpin closure velocity, as the pulling force decreased. We interpret this as an effect of the pressure exerted by the hairpin’s closure on the translocating RecD2, indicating a weak interaction between the protein and the adjacent ssDNA present in the other arm of the hairpin (Fig. 6F).

In summary, these experiments indicate that, at low external force (below 11 pN) or additional factors, RecD2 is not very efficient at unwinding, likely due to its tendency to switch between the strands from unwinding to translocation. This is consistent with the low helicase activity observed in bulk biochemical assays performed in the absence of force (Fig. 3). In our MT experiment, the applied force counteracts the hairpin re-annealing, thereby facilitating its unwinding by RecD2. Indeed, we qualitatively found that the processivity of unwinding was positively related to the magnitude of the applied force. At high forces, the protein appears to prefer positioning facing the duplex, because once the hairpin is fully unwound and begins reforming behind RecD2, the protein tends to switch strands, returning to its preferred orientation for duplex unwinding.

## Discussion

In this study, we characterized both the biochemical properties and single-molecule activities of *B. subtilis* RecD2. Our experiments show that the secondary structure could influence both the binding of RecD2 to ssDNA and the ssDNA-stimulated ATPase activity associated with translocation (Figs 1 and 2). Interestingly, an analogous effect is observed when RecD2 interacts with polydA, a single-stranded polymer with strong base-stacking interactions between adjacent adenines [30]. Additionally, we found that RecD2 exhibits stronger binding to DNA at low MgCl_2_ concentrations (Supplementary Table S5). This aligns with the idea that RecD2 interacts electrostatically with the negatively charged phosphodiester backbone, which becomes shielded at higher MgCl_2_ concentrations. This is consistent with structural data showing Δ150-RecD2_Dra_ interacting with ssDNA primarily through the phosphodiester backbone [13, 40].

On the other hand, we have observed that a 12-mer ssDNA (dT_12_) minimally stimulated the ATP hydrolysis activity of RecD2, which further increased as the length of the poly(dT) DNA was extended. EMSAs showed that RecD2 poorly bound dT_20_ in the presence of the nonhydrolyzable ATP analogue ATPγS, while the binding capacity was increased with the length of the ssDNA. This large binding site size observed here suggests the presence of an additional, unidentified binding locus, as crystal structures show that RecD2 helicases bind a stretch of approximately eight nucleotides [1, 13]. In contrast to our results with the full-length RecD2, previous ATPase assays performed with a RecD2 helicase lacking the first 150 amino acids of the NTD (Δ150-RecD2*_Dra_*) showed minimal variation in ATPase rates with poly(dT) substrates of different lengths (20–60 nt) [13]. These data suggest that the NTD of RecD2 helicases may participate in ssDNA binding. Indeed, biochemical analysis showed that Δ150-RecD2*_Dra_* has less affinity than that of full length RecD2*_Dra_* for a dT_60_ ssDNA [41].

RecD2 helicases share homology with the human helicase HELB, which also features an extended NTD of 467 amino acids preceding its first helicase motif [17]. HELB’s unusually large footprint, spanning 20 nucleotides, along with its ability to form ssDNA loops, suggests the presence of a secondary DNA binding site within its NTD [19]. RecD2 appears to have a similar footprint than HELB, which might also suggest the presence of an additional binding site. Interestingly, the presence of this additional ssDNA binding site is predicted using AlphaFold 3 (Supplementary Fig. S1). However, both proteins behaved differently in single-molecule experiments. We did not observe DNA loop formation by RecD2 in single-molecule translocation assays, nor binding to ssDNA in the absence of ATP. This might be explained by the poor conservation of the NTD of both proteins. In EMSA assays, we detected ssDNA binding in the absence of ATP, although affinity was approximately four to eight times reduced and glutaraldehyde was required to stabilize the protein–DNA complexes (Supplementary Fig. S4). Furthermore, our EMSA assays showed that RecD2 bound with a high affinity to a replication fork but was very sensitive to the nature of the 3′-arm of the fork. When this arm was replicated and was dsDNA, the binding affinity was ∼10 times reduced (Fig. 2 and Supplementary Fig. S6). Together, we hypothesize that despite some differences with HELB, a secondary binding site in the NTD of RecD2 is present and could be important for contacting the other arm of the fork. The importance of the NTD for RecD2 functionality has been highlighted *in vivo* in *Bacillus anthracis*, where a mutant in this region, RecD2*_Ban_* (F208A), exhibited sensitivity to MMC comparable to that of the catalytically inactive mutant RecD2*_Ban_* (K368Q) [9, 42].

RecD2 does not need free ends to bind ssDNA, as the presence of a 5′ or a 3′ tail is not required for the binding of RecD2 on immobilized ssDNA or hairpin tethers. RecD2 translocates processively along ssDNA devoid of secondary structures at a rate of 572 ± 106 nt/s (Fig. 4). These high translocation rates, obtained from optical tweezers experiments, should be considered as the maximum values for RecD2 ATPase turnover, given the expected step size of 1 ATP/base for members of the helicase Superfamily I [13, 43–45]. In analogous single-molecule experiments, the translocation velocity of HELB was measured at 72 ± 40 nt/s [19], approximately eight times slower than RecD2. Due to the similarities between the helicase domains of RecD2 and HELB (both members of the RecD-like family of SF1B helicases), it can be speculated that the differences in translocation speed may arise from structural variations, perhaps in their respective NTDs.

Notably, biochemical ATP hydrolysis assays systematically provided a much lower rate than translocation rates as measured in optical tweezers. This lack of consistency between ATPase rates from bulk and single-molecule assays has been observed before, for example, for *sc*Pif1 [46]. In bulk assays, ATPase rates on short ssDNA oligonucleotides are significantly limited by the protein’s ability to bind to ssDNA. In these experiments, the protein must bind, translocate along the oligonucleotide a few dozen nucleotides at most, and then dissociate. The longer the oligonucleotide, the higher the likelihood that the protein will bind and translocate. This explains the increase in ATPase activity with longer oligonucleotides (Fig. 1A). Therefore, in bulk measurements, we are not directly measuring the ATP consumption strictly coupled to translocation, as the process is constrained by the binding probability. Additionally, bulk measurements provide an average across the entire population, meaning that while some proteins may exhibit higher ATPase rates, others may not be binding the oligonucleotide.

Helicases often interact with other proteins to greatly increase activity. The association of the non-processive helicase PcrA (SF1A) [47] with RepD is a good example, where together can unwinding thousands of base pairs [29]. Association of several helicase monomers that cooperate to increase their activity has also been proposed [39, 48, 49]. Our experiments cannot discard the cooperative action of RecD2 monomers in the unwinding process. Optical tweezers experiments indicated that as protein concentration increased, the overall translocation velocity decreased (Fig. 4D), suggesting that protein crowding on ssDNA hinders translocation. Additionally, the increase of tail length of forked substrates from 37 to 80 nt did not enhance unwinding activity in bulk assays (Fig. 3D). However, we did observe an enhanced unwinding activity in MT experiments at higher protein concentration (Supplementary Fig. S12B). In this case, the higher concentration of proteins may avoid the closure of the hairpin, facilitating its unwinding.

Our biochemical studies have shown that RecD2 exhibits modest helicase activity in bulk assays (Fig. 3C). However, in single-molecule experiments where force is applied to the DNA, unwinding is significantly enhanced. Under these conditions, RecD2 can processively unwind thousands of base pairs when a hybrid DNA is subjected to a force exceeding a threshold of 22 ± 4 pN (Fig. 5) and its unwinding rate can reach 330 ± 100 bp/s at 20–25 pN. Moreover, in the hairpin experiment, where lower forces are applied, RecD2 processively unwinds more than a thousand base pairs at forces over 10 pN. This apparent discrepancy in threshold forces arises from the different manner the force is applied. In the C-Trap unwinding experiment, the force is applied on the translocating strand and this destabilizes base pairing of the duplex at the ss–dsDNA interface. The unwound strand, which is not subjected to force, presumably folds into secondary structures preventing its reannealing with the opposite strand. In the MT hairpin assay, the force is applied at both ends of the hairpin in an unzipping configuration, thus facilitating the separation of both DNA strands [36]. In this case, the force helps in the unwinding activity of RecD2 in that it prevents base pairing by keeping complementary strands at a distance, once the protein has separated them. Overall, these experiments indicate that, in the absence of external force or additional factors, RecD2 is not a processive helicase.

Interestingly, in both single-molecule setups used in this study, we consistently observed strand-switching activity by RecD2. In the C-Trap unwinding experiment, we detected cycles of unwinding and rehybridization, which are compatible with RecD2 translocating along the tensioned strand while displacing complementary DNA (leading to unwinding and to an increase in extension), followed by translocation along the opposite, non-tensioned strand (causing rehybridization and a return to the initial position at the junction) (Fig. 5 and Supplementary Fig. S10). In the MT hairpin experiment, RecD2 unwinds the entire duplex at high forces, detected as an increase in extension, and continues translocating after reaching the hairpin apex. However, shortly after translocating a few tens of nucleotides, and as the hairpin begins to reform behind it (observed as a decrease in extension), RecD2 switches strands and unwinds the partially reformed hairpin, returning the system to its maximum extension once again (Fig. 6). This behavior is characteristic of strand-switching activity, which has been observed in other helicases [37, 38, 50–52] and could be also demonstrated biochemically (Supplementary Fig. S12A).

The highly repetitive DNA strand-switching activity observed for RecD2 suggests that the protein maintains a continuous contact with the ss–dsDNA junction during unwinding. This is supported by our EMSA assays that showed that the affinity of the protein for a 30-nt oligonucleotide is 14 times less than that for a 5′-tailed DNA, that contains an ss–ds junction adjacent to the 30-nt oligonucleotide (Fig. 2). Although the precise strand-switching mechanism is not yet fully understood, it is well established that this process involves the protein interacting with both strands of the DNA junction simultaneously [52, 53], as we observe in our EMSA experiments. Therefore, it is tempting to propose that the large NTD of RecD2, which is structurally located upstream of the helicase motor domains, might play a role in facilitating this strand-switch function. Together, this tendency to switch between the strands of a fork substrate could explain the poor helicase activity observed in bulk biochemical assays (Fig. 3), where RecD2 might engage into a non-productive mode of continuous unwinding and rewinding of a DNA fork. In support of this idea, Alphafold 3 predicts a complex of RecD2 with a fork structure where an additional binding site at the NTD contacts the unwound strand (Supplementary Fig. S13). We propose that modulation of this strand-switching activity either by the application of force or by other protein factors might be relevant for RecD2 function *in vivo*. A similar strand-switching mechanism has been proposed for other helicases. For example, the RecQ HRDC domain is thought to establish a secondary contact with DNA, facilitating RecQ strand-switching activity [54]. RecQ helicases are conserved from prokaryotes to humans, and numerous studies have shown that their interactions with various partners modulate their activity [37, 55, 56]. Similarly, RecD2 unwinding and strand switching may also be influenced by interaction partners. Supporting this idea, some evidence suggests that RecD2 helicases interact with RecA and SSB [11, 57], although further studies are needed to clarify the functional and structural nature of this interaction.

As an accessory helicase in DNA replication, RecD2 may not require extensive processivity, a role primarily handled by the replicative helicase DnaC. Instead, RecD2 has been proposed to function as a fork remodeler, playing a crucial role in maintaining replication fork integrity [11, 58]. Its high affinity for forked substrates found *in vitro* supports its *in vivo* co-localization with replication forks and its suggested role in replication restart [59]. In this context, RecA has been shown to inhibit replication restart by binding to gaps in both the leading and lagging strands [60]. Our work demonstrates that RecD2 can switch between strands, moving both toward and away from the replication fork through its strand-switching activity. This ability likely represents a fundamental mechanism allowing RecD2 to alternate between the leading and lagging strands, removing RecA and facilitating replication restart. Additionally, during recovery of stalled replication forks, a brief unwinding activity is required to allow PriA to bind and activate the DnaC replicative helicase. RecD2’s limited processivity may be well suited for this task.

## Supplementary Material

gkaf459_Supplemental_File

## Data Availability

The data underlying this article are available at Zenodo, https://doi.org/10.5281/zenodo.14749147.
