# Supplementary material for: Bacterial RecD2 is a processive single-stranded DNA translocase with strand-switching capacity at DNA forks
Source: Nucleic Acids Res. 2025 Jun 6;53(11):gkaf459. doi: 10.1093/nar/gkaf459 (PMC12143592; doi:10.1093/nar/gkaf459)
Supplement: gkaf459_Supplemental_File [file gkaf459_supplemental_file.pdf]

Supplementary Materials for

BACTERIAL RECD2 IS A PROCESSIVE SINGLE-STRANDED DNA TRANSLOCASE WITH STRAND-SWITCHING CAPACITY AT DNA FORKS

Silvia Hormeño[1,†], Cristina Ramos[2,†], Javier Mendia-Garcia[1,†], Clara Aicart-Ramos[1], Silvia Ayora[2,*], Fernando Moreno-Herrero[1,*]

[1] Department of Macromolecular Structures, Centro Nacional de Biotecnología, Consejo Superior de Investigaciones Científicas, 28049, Spain.

[2] Department of Microbial Biotechnology, Centro Nacional de Biotecnología, Consejo Superior de Investigaciones Científicas, 28049, Spain.

* To whom correspondence should be addressed. Email: sayora@cnb.csic.es and fernando.moreno@cnb.csic.es

† Joint First Authors

This pdf file includes

Supplementary Methods and References

Figures S1-S13

Tables S1-S5

SUPPLEMENTARY METHODS

DNA Hairpin Substrate Preparation

A 1238 bp-hairpin was fabricated based on a previous design by Manosas et al. (1) with some modifications. The DNA hairpin construct consists of a 1238 bp hairpin with a 4 nt loop (4 dTs), a 31 bp 5′ and 3´-biotinylated labelled dsDNA tail followed by a 45 nt ssDNA segment, and a 146 bp 3′-digoxigenin labelled dsDNA tail. The digoxigenin labeled dsDNA fragment is connected to the hairpin through a 10 nt poly dCs ssDNA region. The dsDNA hairpin was obtained by PCR amplification with Phusion High-Fidelity DNA Polymerase (Thermo Scientific) using Lambda DNA (New England Biolabs) as template and oligonucleotides that include different BsaI restriction sites in each side of the PCR fragment (see **Supplementary Table S3** for oligonucleotides) followed by purification (QIAGEN). This region was selected by running a homemade software that computes the GC content of a given sequence and selecting a running window of 100 bp. After digestion with BsaI restriction enzyme (New England Biolabs) followed by purification, we obtained a dsDNA fragment of 1204 bp with an homogeneous GC content and unique non-palindromic 5´-overhangs. This restriction site was selected to avoid unspecific products of ligation in the later steps. A fork structure was formed by two partially complementary oligonucleotides, the 252.5-Biotin flap, that was 5′-biotinylated for the attachment to the magnetic bead, and the 251.Template hairpin. The two oligonucleotides were annealed by heating at 95°C for 5 min and cooling down to 20°C at a -1°C min^{-1} rate in hybridization buffer (10 mM Tris-HCl pH 8.0, 1 mM EDTA, 200 mM NaCl, 5 mM $MgCl_2$). The final fork structure contained a dsDNA region with a 5´-cohesive end compatible with one of the ends of the PCR fragment. The oligonucleotide 250.Loop hairpin was equally self-annealed to create a short dsDNA hairpin with a cohesive end compatible with the other end of the PCR fragment. The fork structure and the short hairpin oligo (5x excess of each) were overnight ligated to either end of the 1204 bp-PCR fragment by using T4 DNA ligase. Next, the ligated DNA structure was annealed with 10X excess of the biotinylated oligonucleotide 326.3 Biotin anneal 252 short that was complementary to the oligonucleotide 252.5-Biotin flap, and allowed to include an extra biotin in the opposite strand. The oligonucleotide was annealed by heating at 95°C for 1 min and cooling down from 80°C to 10°C at a -0.5°C 10 s^{-1} rate in annealing buffer (10

mM Tris-HCl pH 7.5, 1 mM $MgCl_2$). After hybridization, the DNA construct was gel extracted to remove the excess of oligos, and purified (QIAGEN). Then, it was subsequently annealed with a 5x excess of the oligonucleotide 253.Primer for Dig that was partially complementary through the oligonucleotide 251.Template hairpin, by heating at 95°C for 1 min and cooling down from 80°C to 10°C at a -0.5°C 10 s^{-1} rate in annealing buffer. The digoxigenin label required to attach the DNA hairpin construct to a glass surface via anti-digoxigenin antibodies was then incorporated by filling in the overhangs with Klenow Fragment (3'→5' exo) (New England Biolabs) in the presence of dATP, dCTP and dUTP-digoxigenin (Roche) for 1 h at 37°C followed by heat inactivation for 20 min at 75°C. The temperature was slowly decreased from 75°C to 15°C at a -0.5°C 10 s^{-1} rate to allow reannealing of the oligonucleotide 326.3 Biotin anneal 252 short in case it was detached. The completed DNA hairpin construct was ready to use in MT experiments without further purification. EDTA pH 8.0 to 1 mM final concentration was added to preserve. DNAs were never exposed to intercalating dyes or UV radiation during their production and were stored at 4°C.

SUPPLEMENTARY REFERENCES

1. Manosas,M., Spiering,M.M., Zhuang,Z., Benkovic,S.J. and Croquette,V. (2009) Coupling DNA unwinding activity with primer synthesis in the bacteriophage T4 primosome. *Nat Chem Biol*, **5**, 904–912.
2. Saikrishnan,K., Griffiths,S.P., Cook,N., Court,R. and Wigley,D.B. (2008) DNA binding to RecD: role of the 1B domain in SF1B helicase activity. *EMBO J.*, **27**, 2222–2229.
3. Singleton,M.R., Dillingham,M.S., Gaudier,M., Kowalczykowski,S.C. and Wigley,D.B. (2004) Crystal structure of RecBCD enzyme reveals a machine for processing DNA breaks. *Nature*, **432**, 187–193.

SUPPLEMENTARY FIGURES

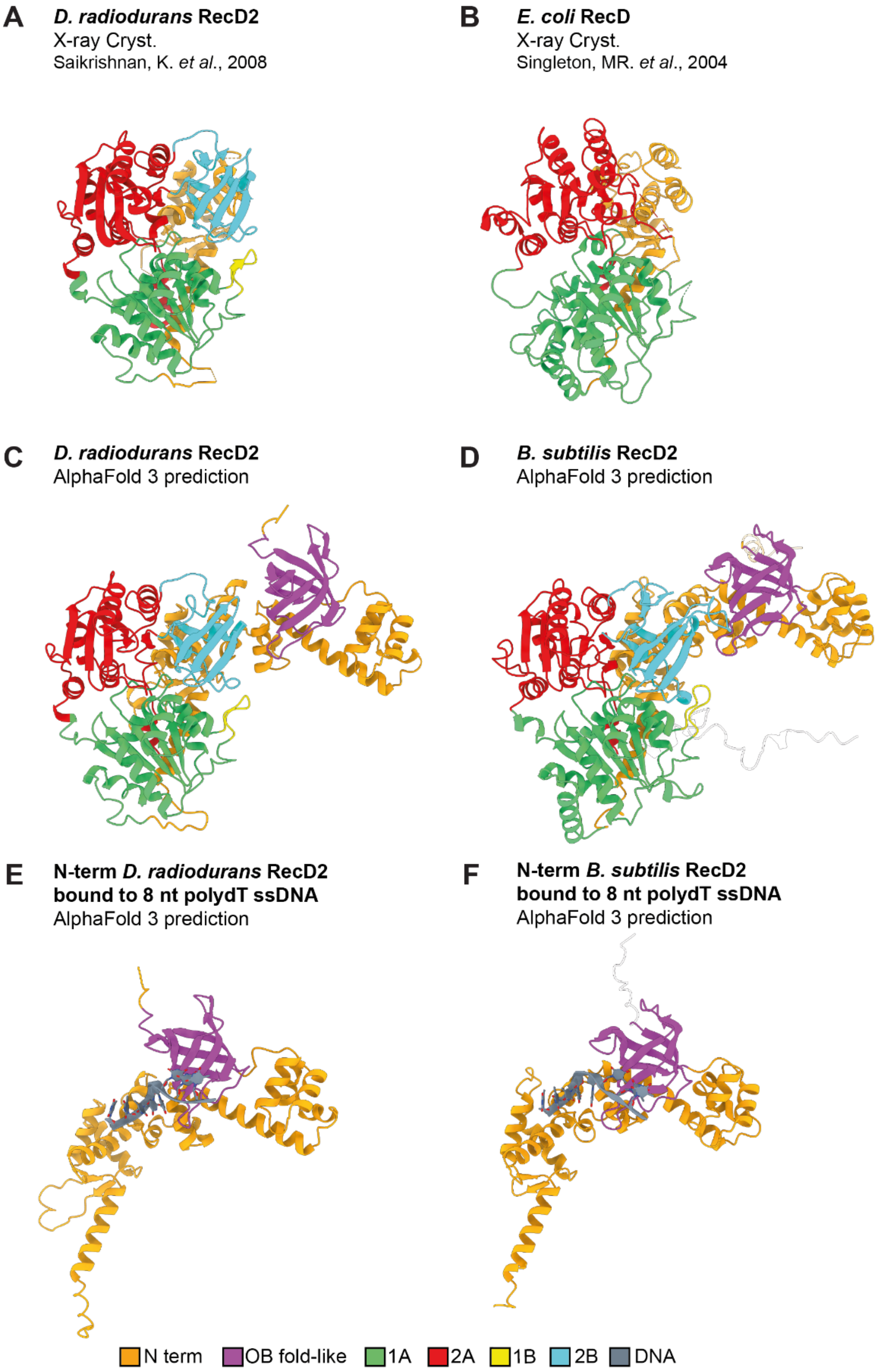

Figure S1. Crystallographic structures and AlphaFold 3 predicted structures of RecD-family helicases. (**A**) Crystallographic structure of a truncated version of *Deinococcus radiodurans* RecD2 lacking the first 150 aa of the N-terminal domain (PDB:

3E1S, named in text as Δ150-RecD2$_{Dra}$) (2). (**B**) Crystallographic structure of *Escherichia coli* RecD (PDB:1W36, a subunit of the RecBCD complex (3). (**C**) AlphaFold 3 prediction of the complete structure of *D. radiodurans* RecD2. An OB-fold like motif (purple) is predicted to be located in the N-terminal domain. (**D**) AlphaFold 3 structure prediction of *Bacillus subtilis* RecD2 showing an OB-fold like motif (purple) in the N-terminal domain. (**E**) and (**F**) AlphaFold 3 predictions of the N-terminal domain of *D. radiodurans* and *B. subtilis* bound to dT_8 (grey), respectively. The OB-fold like motif (purple) is predicted to interact with the DNA. Domain composition range (in aminoacids) was the following. RecD2 (*D. radiodurans*): N-ter 1–329 (OB fold: 5–83), 1A 330–412, 421–495, 1B 413–420, 2A 496–572, 641–715, 2B 573–640. RecD2 (*B. subtilis*): N-ter 1–325 (OB fold: 14–90), 1A 326–433, 443–520, 1B 434–442, 2A 521–604, 684–757, 2B 605–683. RecD (*E. coli*): N-ter 1–132, 1A 133–363, 2A 364–404, 523–606.

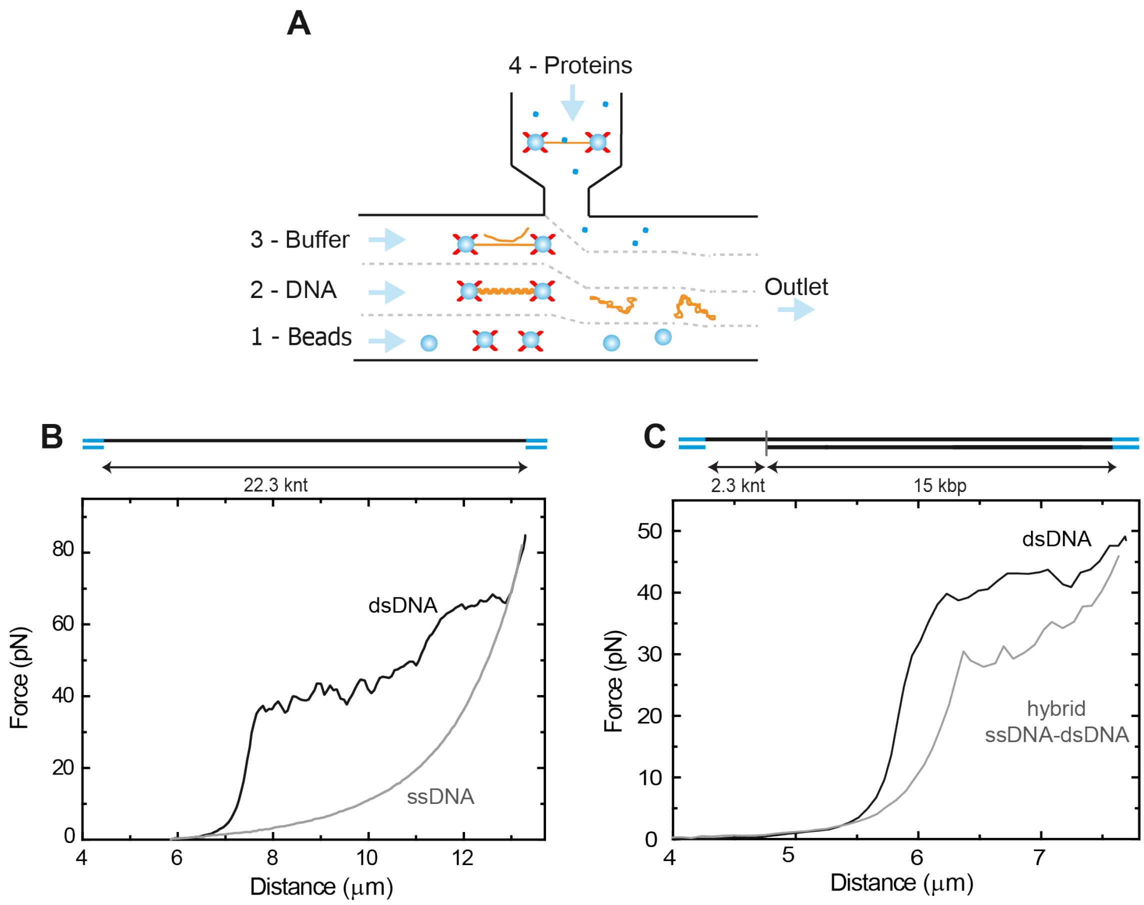

Figure S2. Experimental workflow and ssDNA and ss-dsDNA hybrid *in situ* formation in the C-Trap. (**A**) Schematic of the experimental fluid cell used for optical tweezers experiments. Individual DNA tethers were formed in channels 1-3 separated by laminar flow containing streptavidin-coated beads, DNA molecules for ssDNA or ss-dsDNA formation and TE buffer, respectively. The partial or complete force-induced melting of the duplex takes place in channel 3. After the ssDNA or ss-dsDNA formation, the traps were subsequently moved to channel 4 for protein loading and imaging. (**B**) Force-extension curves indicate the transition from dsDNA (black) to ssDNA (grey). (**C**) Force-extension curves indicate the transition from dsDNA (black) to hybrid ss-dsDNA (grey). Above the curves, schemes of the two DNA constructs are included.

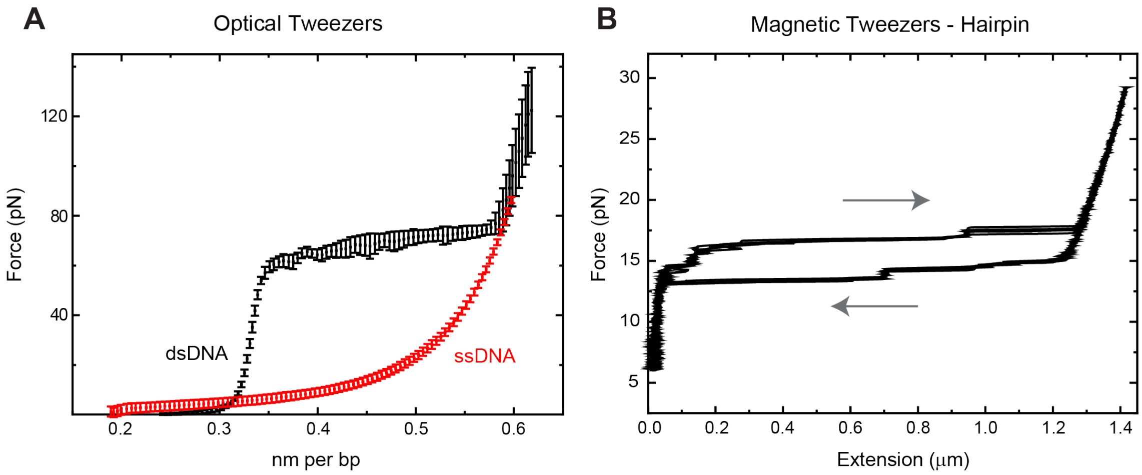

Figure S3. Mechanical properties of dsDNA, ssDNA and the DNA hairpin. (**A**) Averaged force-extension curves of the DNA construct employed for C-Trap experiments. Black points correspond to the stretching curves of 6 dsDNA molecules and red points are from the relaxation curves as ssDNA (N = 8). With applied force, the dsDNA unwinds, transitioning into an ssDNA tether. Since the number of base pairs is known, these experiments enabled us to determine the equivalence between distance and nucleotide. (**B**) Force-induced hairpin unzipping (4 cycles) of the construct used in MT experiments. Increasing the applied force leads first to the entropic stretching of the dsDNA and ssDNA handles of the construct. At 16-17 pN, the 1.2 kbp hairpin unzips and the extension suddenly increases. Then, further gradual stretching is observed. The arrows indicate the stretching and relaxation segments of the force-extension curves.

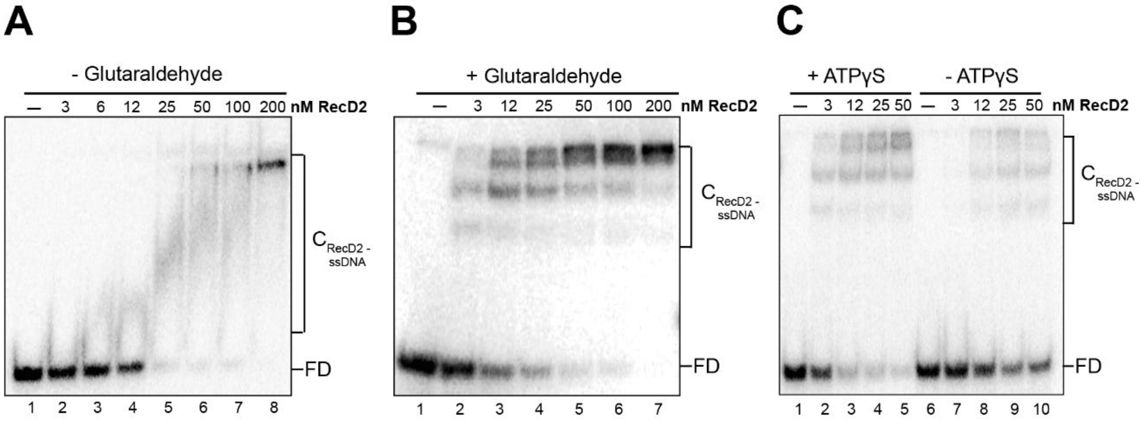

Figure S4. Binding to ssDNA is stimulated by ATPγS and stabilized by glutaraldehyde.

(**A**) Binding in the absence of glutaraldehyde. Increasing amounts of RecD2 were incubated for 15 min at 37ºC with radiolabeled 80 nt ssDNA (0.25 nM in molecules) in a reaction buffer containing 2 mM $MgCl_2$ and 1 mM ATPγS. (**B**) The effect of glutaraldehyde. The radiolabeled 80 nt ssDNA was incubated as in (A). Before electrophoresis 0.05%, (v/v) glutaraldehyde was added and the samples were incubated for 15 min. **(C)** ATPγS stimulates DNA binding. Binding activity of RecD2 to the radiolabeled 80 nt ssDNA in the presence (*lanes 1 to 5*) or in the absence (*lanes 6 to 10*) of 1 mM ATPγS. Complexes were stabilized with 0.05% glutaraldehyde. In all, the samples were separated on 8% polyacrylamide gels, run in 1 X TBE. Protein-DNA complexes were visualized by autoradiography. FD: free DNA. $C_{RecD2\text{-}ssDNA}$: complexes formed due to the binding of RecD2 molecules to the ssDNA.

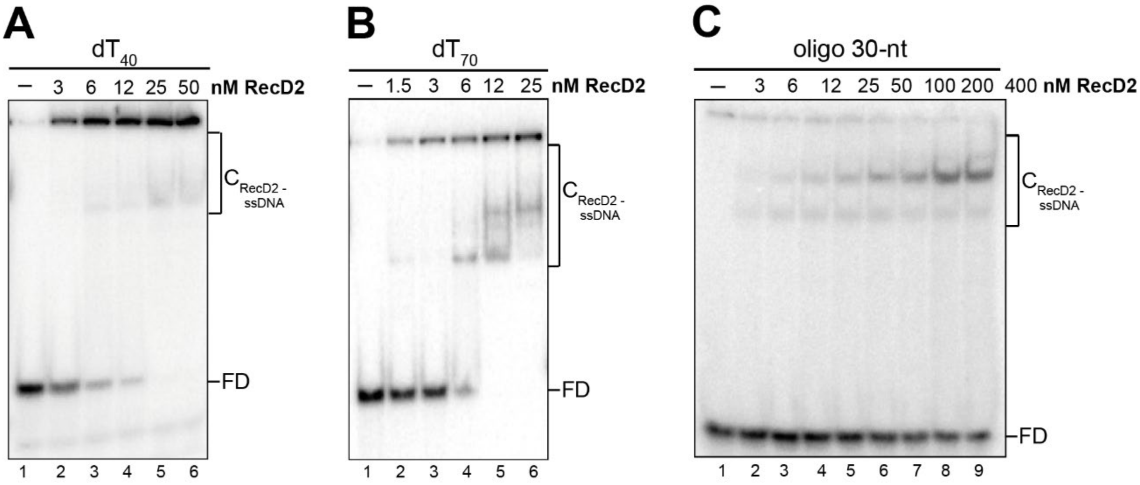

Figure S5. Complementary data to the binding of RecD2 to ssDNA. Increasing amounts of RecD2 were incubated for 15 min at 37ºC with radiolabeled ssDNA (0.25 nM in molecules) in a binding buffer containing 2 mM $MgCl_2$ and 1 mM ATPγS. Then, glutaraldehyde (0.05% v/v) was added, samples were incubated for another 15 min and then separated on 8% PAGE run in 1 X TBE. DNA substrates: dT_{40} (**A**), dT_{70} (**B**). 30 nt-ssDNA (**C**), FD: free DNA. $C_{RecD2\text{-}ssDNA}$: Protein-DNA complexes.

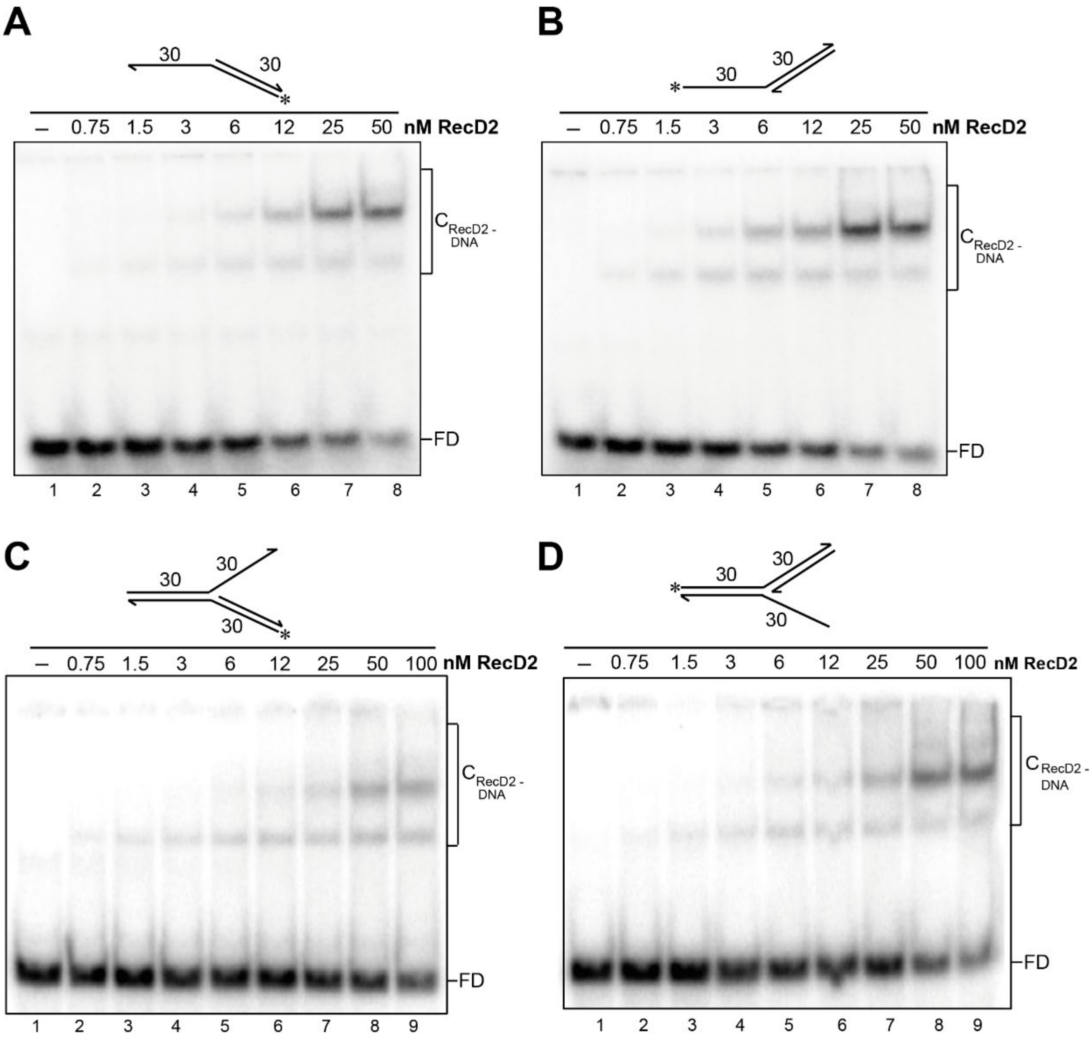

Figure S6. Binding of RecD2 to tailed substrates and tailed replicated forks.

Increasing amounts of RecD2 were incubated for 15 min at 37ºC with radiolabeled ssDNA (0.25 nM in molecules) in a binding buffer containing 2 mM $MgCl_2$ and 1 mM ATPγS. Then, glutaraldehyde 0.05% (v/v) was added, and the samples were separated by 8% PAGE. (**A**) 3′-tailed, (**B**) 5´-tailed, (**C**) 3′-tailed replicated fork, and (**D**) 5′-tailed replicated fork. FD: free DNA. $C_{RecD2\text{-}ssDNA}$: complexes formed due to the binding of RecD2 molecules to the ssDNA.

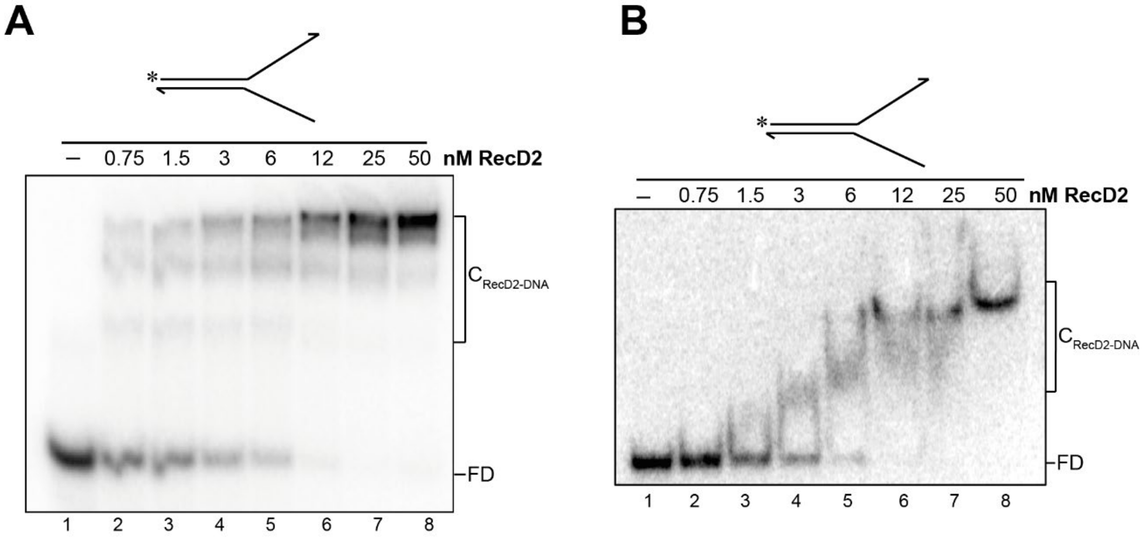

Figure S7. Binding to replication fork structures is stabilized by glutaraldehyde. Radiolabeled fork 30-30 DNA was incubated with increasing concentrations of RecD2 in the presence of 2 mM $MgCl_2$ and 1 mM ATPγS at 37°C. (**A**) Binding to Fork 30-30 in the presence of glutaraldehyde. (**B**) Binding to Fork 30-30 in the absence of glutaraldehyde. Protein-DNA complexes were separated by 8% PAGE. FD: free DNA. $C_{RecD2\text{-}ssDNA}$: Protein DNA complexes.

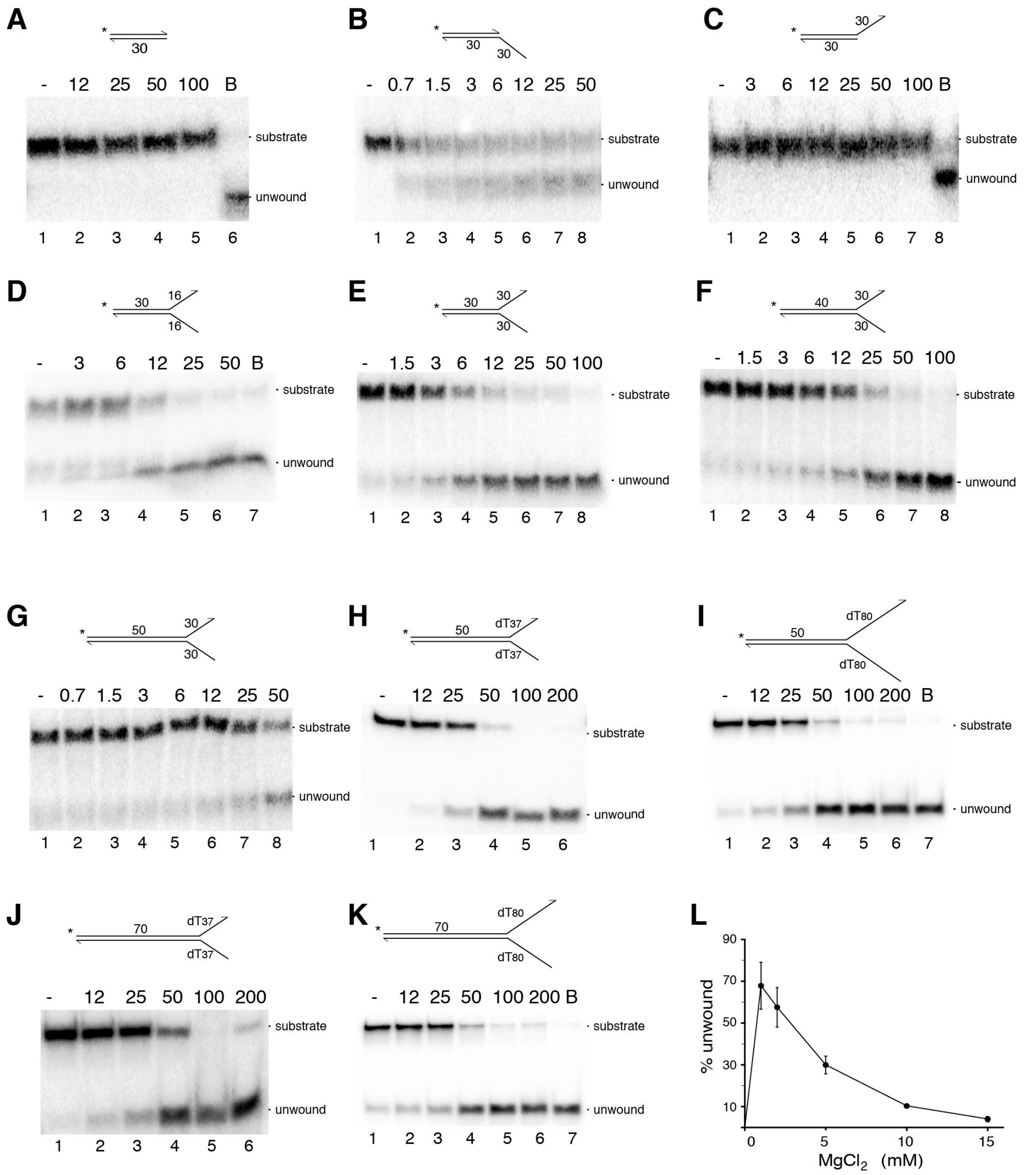

Figure S8. DNA unwinding by RecD2. (**A-K**) 0.25 nM of radiolabeled structures were incubated with increasing concentrations of RecD2 for 15 min at 37ºC in buffer B containing 1 mM ATP and 2 mM $MgCl_2$. Then, reactions were stopped and samples were analyzed by non-denaturing 10% PAGE and autoradiography. Lane B is a marker for the labeled unwound product made by boiling the samples at 100 °C before loading. A representative gel is shown here and compiled data are shown in **Fig. 3**. (**L**) The effect of Mg^{2+} concentration on the unwinding of the Fork 30-30 substrate.

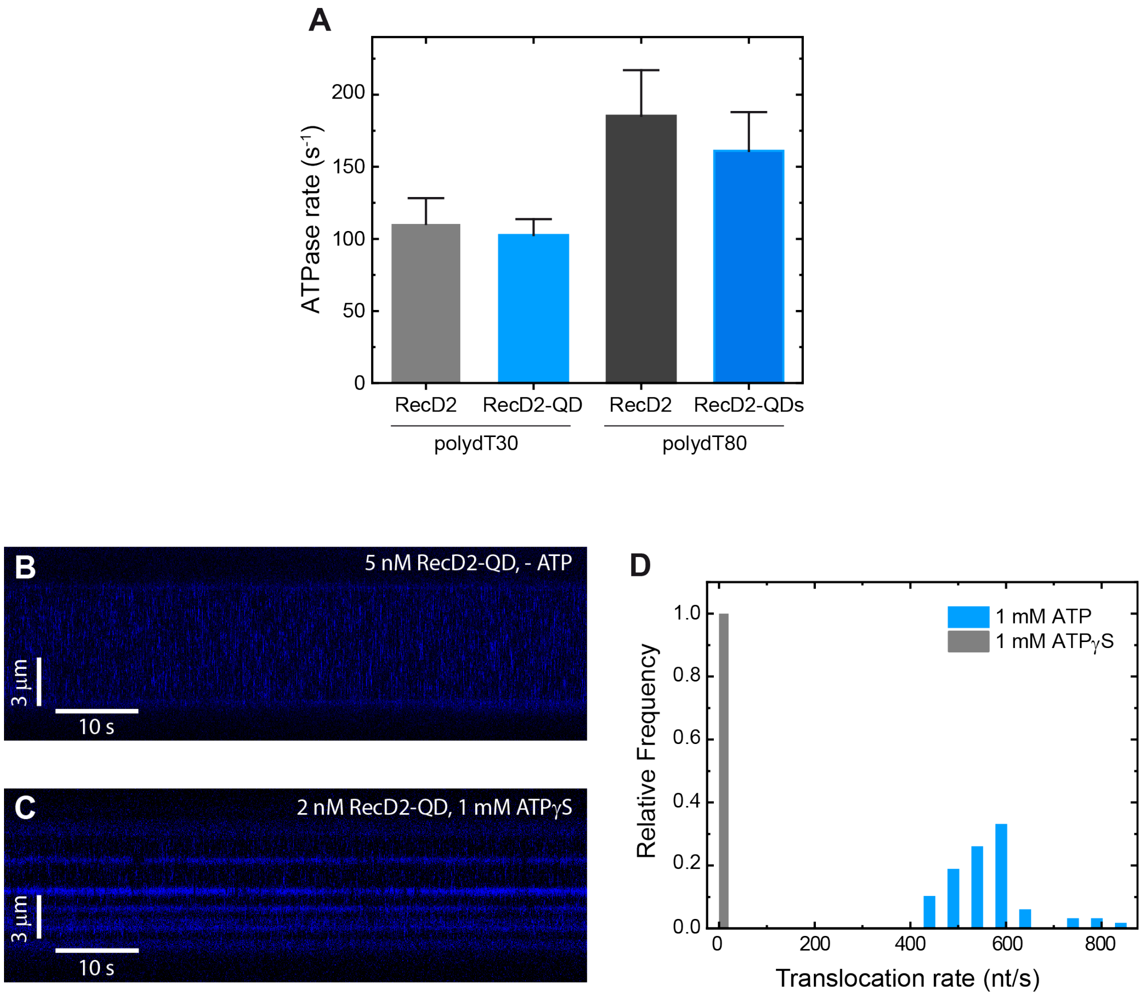

Figure S9. RecD2 requires ATP binding but not hydrolysis to bind ssDNA. RecD2 activity is not affected by QD labelling. (A) ATPase activity of 5 nM RecD2 (grey) and RecD2-QDs (blue) in the presence dT30 and dT80 oligonucleotides (3 µM in nucleotides). **(B)** Representative kymograph showing that RecD2 does not interact with ssDNA in the absence of ATP (F = 15 pN). (**C**) Representative kymograph showing that RecD2 with 1 mM ATPγS has the capability of stably binding ssDNA. Individual trajectories reflect little movement of RecD2 under these conditions (F = 20 pN). (**D**) Distribution of the translocation rate of RecD2 on ssDNA measured from individual trajectories in kymographs obtained in the presence of 1 mM ATP (blue, N = 70) and 1 mM ATPγS (grey, N = 17) (F = 15 and 20 pN). Error bars in (A) are SD of three different experiments.

A

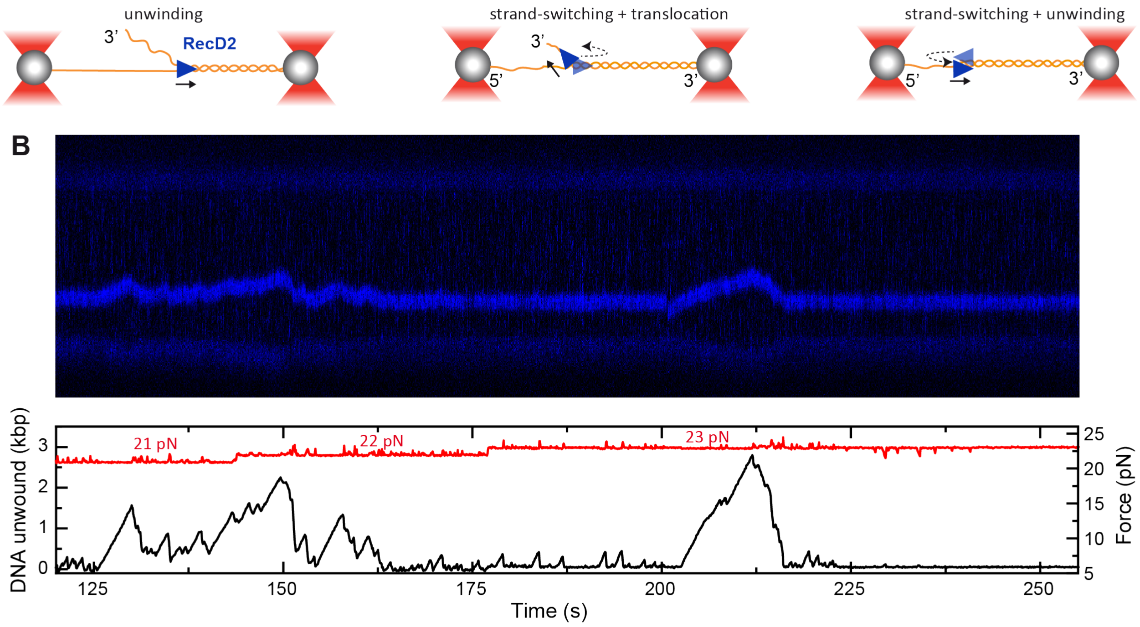

Figure S10. RecD2 strand-switching activity at the ss-dsDNA junction. (**A**) Proposed model of RecD2's strand-switching activity at a DNA fork. During optical tweezers experiments, RecD2 translocates along the strand under tension, displacing the complementary strand. Due to the proximity of the displaced strand, RecD2 can reposition itself onto the opposite strand, leading to helix rewinding behind it, which results in a measured decrease in DNA extension. RecD2 may then fully rewind the duplex DNA region and once it reaches the 3'end pause, undergo another strand switch to initiate a new unwinding cycle on the strand it was originally loaded. (**B**) Representative kymograph showing cycles of unwinding and rewinding by a single fluorescently labeled RecD2 protein on a hybrid DNA construct.

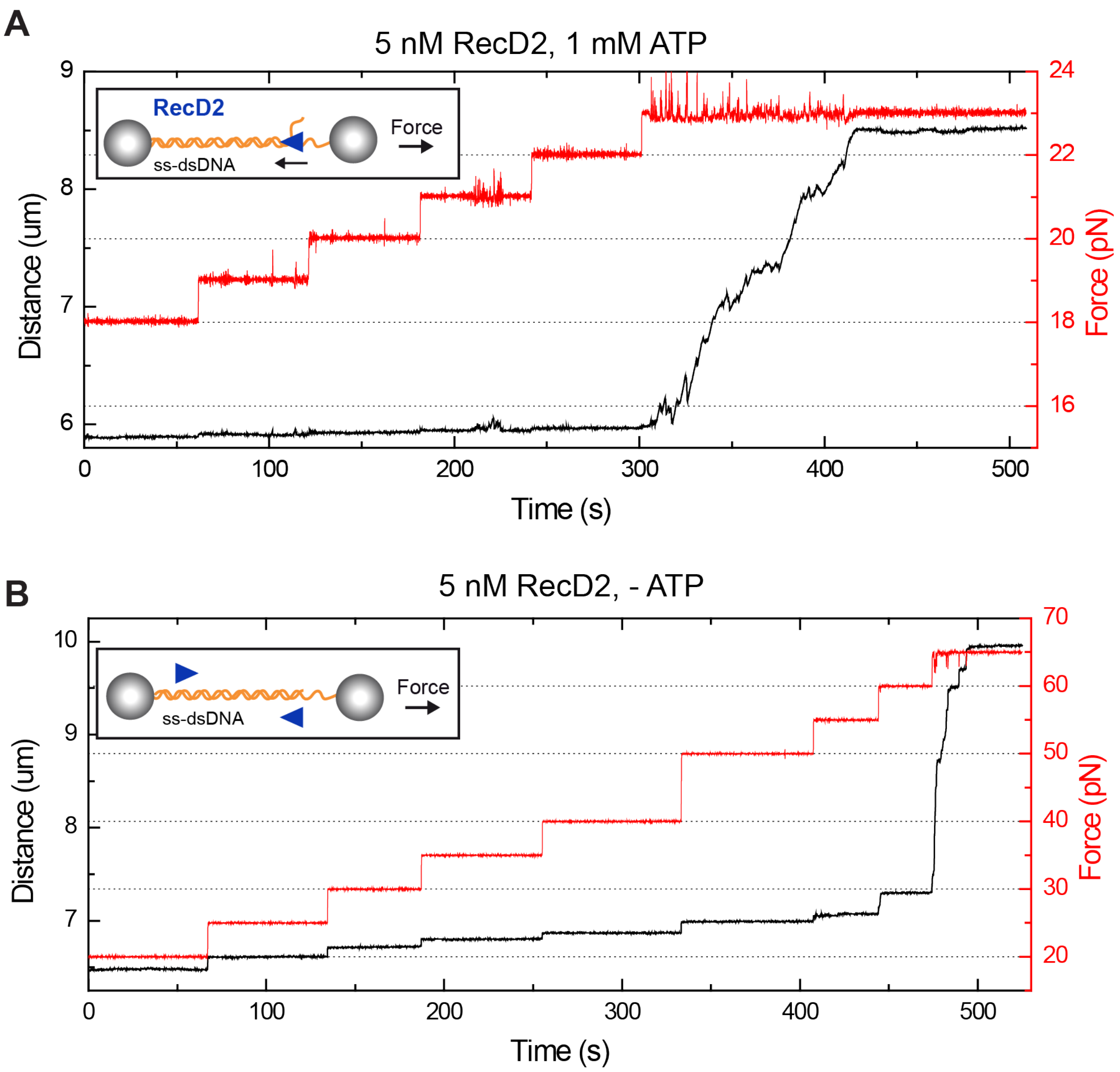

Figure S11. RecD2 unwinds the hybrid DNA only when assisted by force. (**A**) Example of an experiment where the force on the hybrid substrate is increased stepwise in the presence of 5 nM RecD2 and 1 mM ATP. In this case, RecD2 starts to unwind the dsDNA when the force reaches 21 pN and fully unwinds the substrate when force is further increased to 23 pN. (**B**) Control experiment conducted under similar conditions than in (A) but with no ATP. The hybrid DNA is fully unwound when the force is set at 65 pN, the overstretching force. This control demonstrates that RecD2 is responsible of the unwinding during the experiments carried out at forces between 20 and 40 pN.

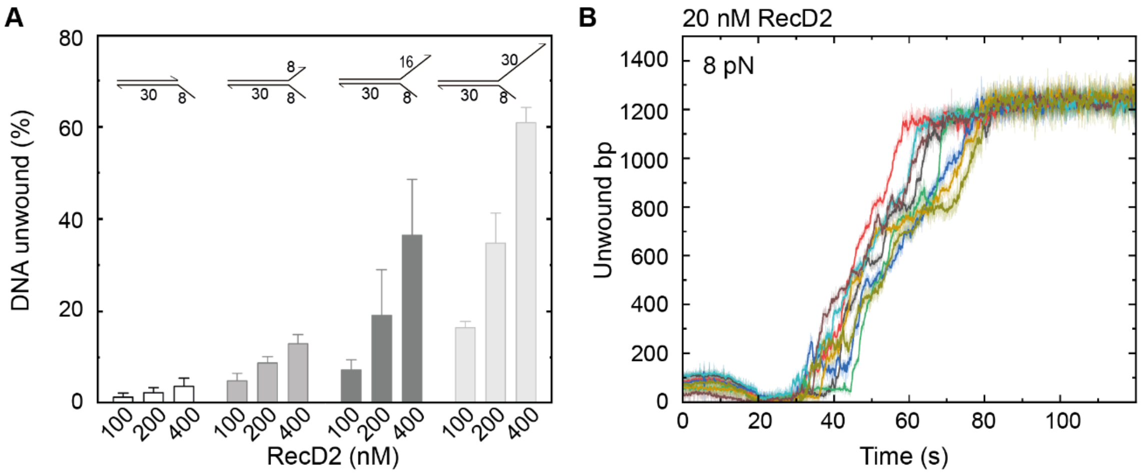

Figure S12. Strand-switching by RecD2 and activity at 20 nM in MT assays. (A) Bulk unwinding experiments to demonstrate the strand-switching activity of RecD2. Used DNA substrates are sketched above each graph. The length of the arms (in nt) are indicated, and the tip of the arrow indicates the 3′-end. In all, 3 nM DNA was incubated with increasing concentrations of RecD2 in the presence of ATP for 15 min at 37ºC. Reaction products were analyzed by native gel electrophoreses and the amount of DNA unwinding determined. The error bars represent the SD of at least three independent experiments. (**B**) RecD2-mediated hairpin unwinding at 8 pN and high protein concentrations.

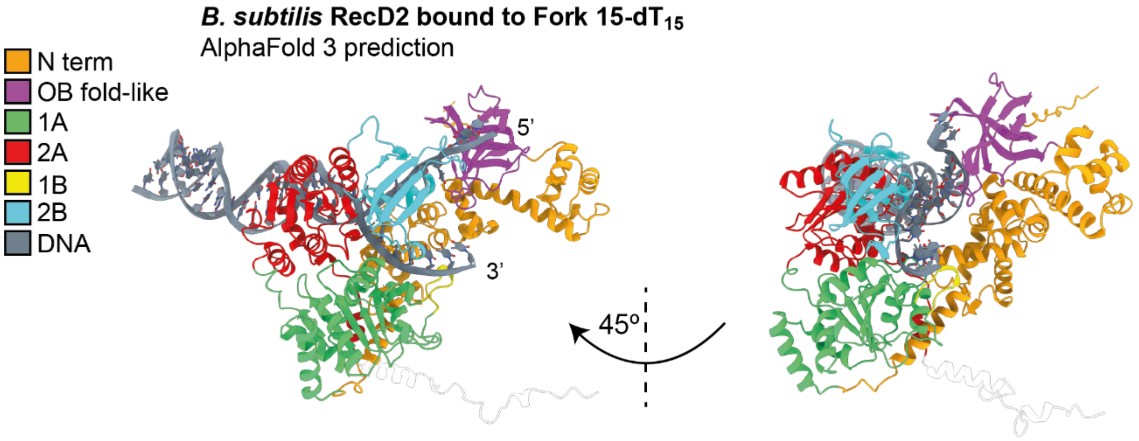

Figure S13. Alphafold3 models of RecD2 bound to Fork 15-dT$_{15}$. The model shows how the NTD and motor domains of RecD2 may contact both DNA tails of a 15-dT$_{15}$ forked DNA in a translocating configuration. We hypothesize that the strand switching capacity of RecD2 is caused by its ability to bind DNA through a secondary binding site at the NTD. Colored regions correspond to different domains as indicated in the legend.

Table S1. Oligonucleotides used in bulk assays

Name	Sequence (5′ to 3′)
**poly(dT)$_8$**	TTTTTTTT
**poly(dT)$_{12}$**	TTTTTTTTTTTT
**poly(dT)$_{15}$**	TTTTTTTTTTTTTTT
**poly(dT)$_{20}$**	TTTTTTTTTTTTTTTTTTTT
**poly(dT)$_{30}$**	TTTTTTTTTTTTTTTTTTTTTTTTTTTTTT
**poly(dT)$_{40}$**	TTTTTTTTTTTTTTTTTTTTTTTTTTTTTTTTTTTTTTTT
**poly(dT)$_{60}$**	TTTTTTTTTTTTTTTTTTTTTTTTTTTTTTTTTTTTTTTTTTTTTTTTTTTTTTTTTTT<br>T
**poly(dT)$_{70}$**	TTTTTTTTTTTTTTTTTTTTTTTTTTTTTTTTTTTTTTTTTTTTTTTTTTTTTTTTTTT<br>TTTTTTTTTTT
**poly(dT)$_{80}$**	TTTTTTTTTTTTTTTTTTTTTTTTTTTTTTTTTTTTTTTTTTTTTTTTTTTTTTTTTTT<br>TTTTTTTTTTTTTTTTTTTTT
**poly(dA)$_{80}$**	AAAAAAAAAAAAAAAAAAAAAAAAAAAAAAAAAAAAAAAAAAAAAAAA<br>AAAAAAAAAAAAAAAAAAAAAAAAAAAAAAAA
**(dT)$_{30}$-60 up**	ACGGCCTAGATCGTGAACCTGCAGGTAAGGTTTTTTTTTTTTTTTTTTTTTTT<br>TTTTTTT
**(dT)$_{30}$-60 down**	TTTTTTTTTTTTTTTTTTTTTTTTTTTTTTCCTTACCTGCAGGTTCACGATCTAG<br>GCCGT
**17-M-38**	ACGGCCTAGATCGTGAACCTGCAGGTAAGGGGCTGCTCA
**17-M-46**	ACGGCCTAGATCGTGAACCTGCAGGTAAGGGGCTGCTCATCGTAGG
**17-M-60**	ACGGCCTAGATCGTGAACCTGCAGGTAAGGGGCTGCTCATCGTAGGTTA<br>GCGTAAATTAG
**17-M-70**	ACGGCCTAGAACGGCCTAGATCGTGAACCTGCAGGTAAGGGGCTGCTCA<br>TCGTAGGTTAGCGTAAATTAG
**17-M-80**	ACGGCCTAGAACGGCCTAGAACGGCCTAGATCGTGAACCTGCAGGTAAG<br>GGGCTGCTCATCGTAGGTTAGCGTAAATTAG
**18-M-38**	GTAGGATACCTTACCTGCAGGTTCACGATCTAGGCCGT
**18-M-46**	CATCAGTCGTAGGATACCTTACCTGCAGGTTCACGATCTAGGCCGT
**18-M-60**	GTGCCTTGCTAGGACATCAGTCGTAGGATACCTTACCTGCAGGTTCACGA<br>TCTAGGCCGT
**18-M-70**	GTGCCTTGCTAGGACATCAGTCGTAGGATACCTTACCTGCAGGTTCACGA<br>TCTAGGCCGTTCTAGGCCGT
**18-M-80**	GTGCCTTGCTAGGACATCAGTCGTAGGATACCTTACCTGCAGGTTCACGA<br>TCTAGGCCGTTCTAGGCCGTTCTAGGCCGT
**21-M-30**	TATCCTACGACTGATGTCCTAGCAAGGCAC

**22-M-30**	CTAATTTACGCTAACCTACGATGAGCAGCC
**KpnI-30**	GTACCTCTAGAGTCGACCTGCAGGCATGCA
**KpnI-50**	GTACCGCCAGTACTAAGCTTCGATTTCTAGAGTCGACCTGCAGGCATGCA
**HindIII-35**	AGCTTGCATGCCTGCAGGTCGACTCTAGAGGTACC
**HindIII-55**	AGCTTGCATGCCTGCAGGTCGACTCTAGAAATCGAAGCTTAGTACTGGCG GTACC
**20-(dT)$_{37}$**	AGCTGCTCATCGTAGGCTAGTTTTTTTTTTTTTTTTTTTTTTTTTTTTTTTTTTT TT
**16-(dT)$_{37}$**	TTTTTTTTTTTTTTTTTTTTTTTTTTTTTTTTTTTTTCTAGCCTACGATGAGC
**20-(dT)$_{80}$**	AGCTGCTCATCGTAGGCTAGTTTTTTTTTTTTTTTTTTTTTTTTTTTTTTTTTTT TTTTTTTTTTTTTTTTTTTTTTTTTTTTTTTTTTTTTTTTTTTTT
**16-(dT)$_{80}$**	TTTTTTTTTTTTTTTTTTTTTTTTTTTTTTTTTTTTTTTTTTTTTTTTTTTTTTTTTTTT TTTTTTTTTTTTTTTTTTTTCTAGCCTACGATGAGC
**Cy5-17M30**	[Cyanine5]ACGGCCTAGATCGTGAACCTGCAGGTAAGG
**Cy5-17M38**	[Cyanine5]ACGGCCTAGATCGTGAACCTGCAGGTAAGGTTTTTTTT
**Cy5-17M46**	[Cyanine5]ACGGCCTAGATCGTGAACCTGCAGGTAAGGGGCTGCTCATCGT GG
**Cy5-17M60**	[Cyanine5]ACGGCCTAGATCGTGAACCTGCAGGTAAGGGGCTGCTCATCG TAGGTTAGCGTAAATTAG
**8T30**	TTTTTTTTCCTTACCTGCAGGTTCACGATCTAGGCCGT

Table S2. DNA substrates used in bulk assays

Name	Oligonucleotide composition	Structure
**5'-tailed**	17-M-60 + 22-M-30	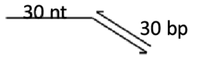
**3'-tailed**	18-M-60 + 21-M-30	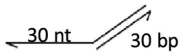
**Fork 30-30**	17-M-60 + 18-M-60	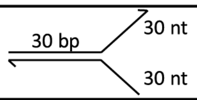
**5'-tailed replicated fork**	17-M-60 + 18-M-60 + 22-M-30	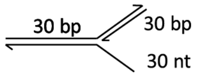
**3'-tailed replicated fork**	17-M-60 +18-M-60 + 21-M-30	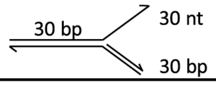
**Fork 30-8**	17-M-38 + 18-M-38	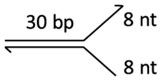
**Fork 30-16**	17-M-46 + 18-M-46	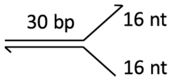
**Fork 30-dT$_{30}$**	dT$_{30}$-mer up + dT$_{30}$-mer down	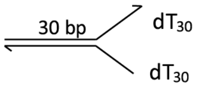
**Fork 40-30**	17-M-70 + 18-M-70	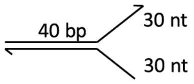
**Fork 50-30**	17-M-80 + 18-M-80	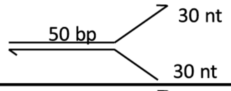
**Fork 50-dT$_{37}$**	KpnI-30 + HindIII-35 + 20-dT$_{37}$ + 16-dT$_{37}$	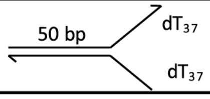
**Fork 70-dT$_{37}$**	KpnI-50 + HindIII-55 + 20-dT$_{37}$ + 16-dT$_{37}$	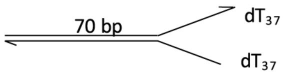
**Fork 50-dT$_{80}$**	KpnI-30 + HindIII-35 + 20-dT$_{80}$ + 16-dT$_{80}$	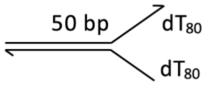
**Fork 70-dT$_{80}$**	KpnI-50 + HindIII-55 + 20-dT$_{80}$ + 16-dT$_{80}$	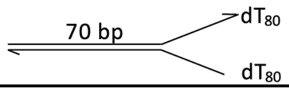
**5'-tailed-8nt**	Cy5-17M30 + 8T30	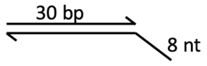
**Fork 8-8**	Cy5-17M38 + 8T30	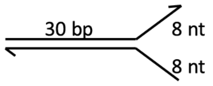
**Fork 16-8**	Cy5-17M46 + 8T30	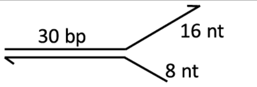
**Fork 30-8**	Cy5-17M60 + 8T30	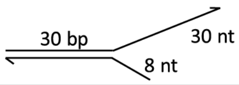

Table S3. Oligonucleotides employed to fabricate the DNA Hairpin Substrate

Fragment	Name	Sequence (5´ to 3´)
**PCR Hairpin fragment**	**248.F-Lambda BsaI 40037**	GCGTAAGTGGTCTCACCGAGCACTACTGGCTGGTTACCAA<br>C
	**249.R-Lambda BsaI 41236**	GCTTCCATGGTCTCATTACCACAACTTCCCTGACAAACCG
**Biotinylated fork structure**	**251.Template hairpin**	[Pho]GTAACCTGTAGCTATATGTCTCCGCCCCCCCCCCTGT<br>GTGTGTGTGTGGTTGTGTGGTGTGTGGTTGTGTGTTGGTG<br>GTTGCATACTTCCGGGAACGCAG
	**252.5-Biotin flap**	[biotin]AATTGCATGTATTACTTGGTAGGATCCGTCATAGC<br>TTTAGCGATTTGGGACACTTCATCAAGACTTCCAGAGCAG<br>CCGGAGACATATAGCTACAGG
**Short hairpin**	**250.Loop hairpin**	[Pho]TCGGGTCAGATGCCTTTTGGCATCTGAC
**To create the biotinylated labelled dsDNA tail**	**326.3 Biotin anneal 252 short**	TGACGGATCCTACCAAGTAATACATGCAATT[biotin-TEG]
**For Dig labelling**	**253.Primer for Dig**	AAAAAAGTGTTGTGTGGTGTTGTTTGGGTGTTGTTTGTGTG<br>TTGTTTGGTGTTGTTTGGGTGTTGTTTGTGTGTTGTTTGCTG<br>CGTTCCCGGAAGTATGC

Table S4. Sequence of the dsDNA central part of the final Hairpin Substrate

Size (bp)	Sequence (5´ to 3´)
**1238 + 4 dTs (red)**	CGGAGACATATAGCTACAGGTTACCACAACTTCCCTGACAAACCGATATGTCATTGG<br>ATGTTCACAACCTGTATCCATGAAAACGTAATGCACGTCTTTACCTGCCCGTCGCTTT<br>TGCTCCATTAGCCAGAGCAAATATGCTGACGTCCTGCCACCGGAGAAACTAACGAC<br>ATTTATCATGCAGCCCTGTCTCCCCATCTCGCTTTCCACTCCAGAGCCAGTCTCGCTT<br>CGTCTGACCACTTAACGCCACGCTCTGTACCGAATGCCTGTATAAGCTCTAATAGCT<br>CCGCAAATTCGCCTACACGCATCCTGCTGGTTGACTGGCCTATTACCACAAAGCCAT<br>TCCCGGCAAGGTTAGGAACAACATCCTGCTGCTTTAATGCTGCGGTAAACACACACT<br>TCCAGCTTTCTGCATCCAGCCAGCGACCATGCCATTCAACCTGACGAGAGACGTCAC<br>CTAAGCAGGCCCATAGCTTCCTGTTTTGGTCTAAGCTGCGGTTGCGTTCCTGAATGG<br>TTACTACGATTGGTTTGGTTGGGTCTGGAAGGATTTGCTGTACTGCGTGAATAGCGTT<br>TTGCTGATGTGCTGGAGATCGAATTTCAAAGGTTAGTTTTTTCATGACTTCCCTCTCC<br>CCCAAATAAAAAGGCCTGCGATTACCAGCAGGCCTGTTATTAGCTCAGTAATGTAGA<br>TGGTCATCTTTTAACTCCATATACCGCCAATACCCGTTTCATCGCGGCACTCTGGCGA<br>CACTCCTTAAAAACCAGGTTCGTGCTCATCTTTCCTTCCCGTTCTTCCCTGGTAGCAA<br>ACCGGTAATACACCGTTCGCCAGACCTTACCTTCGATAACCAGAAGACCTGCCCGT<br>GCCATTTTAGCCGCGGCCTGATTTATGCTGGTTACTGTTGCGCCTGTTAGCGCGGCA

	ACGTCCGGCGCACAGAAGCTATTATGCGTCCCCAGGTAATGAATAATTGCCTCTTTG CCCGTCATACACTTGCTCCTTTCAGTCCGAACTTAGCTTTGATTTCTGCGATCTTCGC CAGAGCCTGTGCACGATTTAGAGGTCTACCGCCCATGACAGGAAGTTGTTTTACTGG TTCAGGGATCGCCTCACCACGGTTAATTCTCGCAGTCATATGGACAAGCTCATCTGC GGCCTTACGGCGTAATTCCGCATCAGTAAGCGCATTGGCCCGCATGTTCTGATACAG GTTGGTAACCAGCCAGTAGTGCTCGGGTCAGATGCCTTTT

Table S5. Effect of $MgCl_2$ in binding to ssDNA

ssDNA with random sequence	***Kapp*** **2 mM $MgCl_2$**	***Kapp*** **10 mM $MgCl_2$**
**30 nt** (22-M-30)	212 ± 17.7	246 ± 20.2
**60 nt** (17-M-60)	13.5 ± 4.2	44.2 ± 14.1
**80 nt** (17-M-80)	11.1 ± 5.4	39.1 ± 15.4

The apparent binding constants K_{app} (in nM), which represent the concentration of RecD2 that binds 50% of the DNA, are shown as mean ± SD for at least three independent experiments. In brackets, it is stated the oligonucleotide used (Table S1)
